# Unified method to integrate and blend several, potentially related, sources of information for genetic evaluation

**DOI:** 10.1186/s12711-014-0059-3

**Published:** 2014-09-30

**Authors:** Jérémie Vandenplas, Frederic G Colinet, Nicolas Gengler

**Affiliations:** University of Liege, Gembloux Agro-Bio Tech, 5030 Gembloux, Belgium; National Fund for Scientific Research, 1000 Brussels, Belgium

## Abstract

**Background:**

A condition to predict unbiased estimated breeding values by best linear unbiased prediction is to use simultaneously all available data. However, this condition is not often fully met. For example, in dairy cattle, internal (i.e. local) populations lead to evaluations based only on internal records while widely used foreign sires have been selected using internally unavailable external records. In such cases, internal genetic evaluations may be less accurate and biased. Because external records are unavailable, methods were developed to combine external information that summarizes these records, i.e. external estimated breeding values and associated reliabilities, with internal records to improve accuracy of internal genetic evaluations. Two issues of these methods concern double-counting of contributions due to relationships and due to records. These issues could be worse if external information came from several evaluations, at least partially based on the same records, and combined into a single internal evaluation. Based on a Bayesian approach, the aim of this research was to develop a unified method to integrate and blend simultaneously several sources of information into an internal genetic evaluation by avoiding double-counting of contributions due to relationships and due to records.

**Results:**

This research resulted in equations that integrate and blend simultaneously several sources of information and avoid double-counting of contributions due to relationships and due to records. The performance of the developed equations was evaluated using simulated and real datasets. The results showed that the developed equations integrated and blended several sources of information well into a genetic evaluation. The developed equations also avoided double-counting of contributions due to relationships and due to records. Furthermore, because all available external sources of information were correctly propagated, relatives of external animals benefited from the integrated information and, therefore, more reliable estimated breeding values were obtained.

**Conclusions:**

The proposed unified method integrated and blended several sources of information well into a genetic evaluation by avoiding double-counting of contributions due to relationships and due to records. The unified method can also be extended to other types of situations such as single-step genomic or multi-trait evaluations, combining information across different traits.

**Electronic supplementary material:**

The online version of this article (doi:10.1186/s12711-014-0059-3) contains supplementary material, which is available to authorized users.

## Background

Simultaneous use of all available data by best linear unbiased prediction (BLUP) is a condition to predict unbiased estimated breeding values (EBV) [1]. However, this condition is not often fully met. For example, in dairy cattle, while foreign bulls are often widely used, e.g. through artificial insemination, evaluating populations based only on internal phenotypic data (i.e. internal records) will lead to potentially biased and less accurate evaluations [2]. The reason is that external phenotypic data used to select these foreign bulls are not available at the internal level. Multiple across country evaluation (MACE), performed at an international level by International Bull Service (Interbull, Uppsala, Sweden), allows EBV, for each population scale, to be aggregated into a single ranking for international dairy sires. However, this has no influence on internal evaluations. These issues are also relevant in the setting of current developments of genomic multi-step or single-step prediction methods (e.g., [3-5]).

Because external phenotypic data are not available at the internal level, methods were developed to combine external information, i.e. external EBV and associated reliabilities (REL), with internal data to improve accuracy of internal genetic evaluations. A first type of approaches is based on performing, a posteriori, an additional step after the genetic evaluation at the internal level. These approaches combine external and internal EBV based on selection index theory (e.g., [6]), based on mixed model theory (e.g., [7]) or based on bivariate evaluations (e.g., [8]). One of the problems of a posteriori approaches is that external information used for selection will not contribute to the estimation of fixed effects at the internal level, which can create potential biases. A second type of approaches combines external information simultaneously with internal phenotypic data in genetic evaluations at the internal level. Simultaneous combination of external information and internal phenotypic data can be carried out using different methods. However, to our knowledge, the following two approaches are the most used. First, external information can be directly included by converting this information into pseudo-records for fictive daughters of external animals (e.g., [2]). Similar approaches were proposed to include external information into internal single-step genomic evaluations (e.g., [5,9]). Second, external information can be directly included by changing both the mean and (co)variance of the prior distributions of genetic effects in a Bayesian approach, as mentioned, for example, by Gianola and Fernando [10]. Quaas and Zhang [11,12] and Legarra et al. [13] proposed two Bayesian derivations to integrate external information into internal genetic evaluations in the context of multi-breed genetic evaluations for beef cattle. These two derivations consider external information as priors of internal genetic effects. Vandenplas and Gengler [14] compared these two derivations and proposed some improvements that concerned mainly double-counting of contributions due to relationships among external animals. Indeed, an EBV of an animal combines information from its own records (i.e., contributions due to own records) and from records of all relatives through its parents and its progeny (i.e., contributions due to relationships) [6,15]. Therefore, integration of EBV for relatives can cause the same contributions that are due to relationships to be counted several times, which can bias genetic evaluations at the internal level.

Both types of approaches i.e. that combine available information a posteriori or simultaneously, raise another issue if the external information results from an evaluation that combines external and internal records, which is that some contributions due to records will be considered several times when external information is combined with internal records. Although this is a major issue for common sources of external information (e.g., MACE information), to our knowledge, only a few studies have proposed solutions to the double-counting of contributions due to records (e.g., [5,16,17]). The proposed solutions were developed as an additional pre-processing step before integration of external information. Furthermore, in many situations, integration of several sources of external information into genetic evaluations at the internal level may be needed but this has not been studied to our knowledge. In such cases, double-counting of contributions due to records could be worse if external information from several evaluations were, at least partially, based on the same internal records, and/or on the same external records, and integrated into the same genetic evaluation.

Thus, the aim of this research was to develop a unified method to integrate and blend simultaneously several, potentially related, external sources of information into an internal genetic evaluation based on a Bayesian approach. In order to achieve this aim, methods were developed to avoid double-counting of contributions due to relationships and due to records generated by the integration of several sources of information. This resulted in modified mixed model equations (MME) that integrate and blend simultaneously several sources of information and avoid double-counting of contributions due to relationships and due to records. The performance of the developed equations was evaluated using simulated and real datasets.

## Methods

### Integration of several sources of external information

Assume an internal genetic evaluation (referred to with the subscript E_0_) based on internal data (i.e. a set of phenotypic records: ${\mathbf{y}}_{{\mathrm{E}}_0}$) that provides internal information (i.e. EBV and associated REL obtained from the evaluation E_0_). Also, assume an i^th^ external genetic evaluation (*i = 1, 2, …, N*, referred to with the subscript E_i_) that is based on the i^th^ source of external data (i.e. the i^th^ set of phenotypic records not used by evaluation E_0_ and free of internal data: ${\mathbf{y}}_{{\mathrm{E}}_{\mathrm{i}}}$) and that provides the i^th^ source of external information, i.e., all available external EBV (EBV_Ei_) and associated REL (e.g., EBV and associated REL obtained from evaluation E_1_ based only on external data E_1,_ and EBV and associated REL obtained from evaluation E_2_ based only on external data E_2_). In addition to be free of internal data, it is also assumed that each i^th^ source of external data was free of the other *N*-1 sources of external data. These assumptions lead to each i^th^ source of external information to be free of internal data and information, as well as of the *N*-1 other external data and information.

Two groups of animals, hereafter called external and internal animals, are defined according to the i^th^ source of external information. Therefore, for each i^th^ source of external information, external animals (subscript A_i_ with *i* = 1, 2,…, *N*) are defined as animals that are associated with this i^th^ source of external information and for which internal data and/or information is available or that have relationships with animals involved in the internal evaluation E_0_. All animals that are not defined as external animals for the i^th^ source of external information are defined as internal animals (subscript ${\mathrm{A}}_{\mathrm{i}}^0$). Internal animals are then defined as animals associated with only internal information when considering the i^th^ source of external information. It is noted that external animals may be associated with different sources of external information and that an animal may be considered as external for the i^th^ source of external information and internal for the *N*-1 other sources of external information because the definitions of external and internal animals depend only on the source of external information considered. Those definitions are summarized in Table 1. In addition, because pedigree information for animals can be easily integrated into a genetic evaluation, it is assumed that the same complete pedigree information could be used for all animals for each genetic evaluation. Concerning the notation of matrices in the following sections (e.g., ${\mathbf{X}}_{{\mathrm{E}}_{\mathrm{i}}\left({\mathrm{A}}_{\mathrm{l}}\right)}$), the subscript E_i_ refers to the i^th^ source of external information and the subscript within brackets (A_l_) refers to the l^th^ group of animals.

**Table 1:** **Concepts related to the terminology of internal and external animals and information**

**Data** ^**1**^	**Pedigree**	
	**Internal animals**	**External animals**
Internal data	Internal evaluation	Internal - external evaluations
	Internal information^2^	Internal - external information
External data	-	External evaluation
		External information

^1^Data = set of phenotypic records; ^2^Information = estimated breeding values and associated reliabilities.

The *N* sources of external information must be integrated into the internal evaluation E_0_. For external animals associated with the i^th^ source of external information, all EBV_Ei_ are summarized by the vector of external EBV, ${\widehat{\mathbf{u}}}_{{\mathbf{E}}_{\mathbf{i}}\left({\mathbf{A}}_{\mathbf{i}}\right)}$, and by the prediction error (co)variance matrix, ${\mathbf{D}}_{{\mathrm{E}}_{\mathrm{i}}\left({\mathrm{A}}_{\mathrm{i}}\right)}$. Because ${\widehat{\mathbf{u}}}_{{\mathrm{E}}_{\mathrm{i}}\left({\mathrm{A}}_{\mathrm{i}}\right)}$ could be estimated with an equivalent external genetic evaluation that includes the internal animals in the pedigree through a genetic (co)variance matrix extended to all animals for the i^th^ source of external information, $$ {\mathbf{G}}_{{\mathrm{E}}_{\mathrm{i}}}=\left[\begin{array}{cc}\hfill {\mathbf{G}}_{{\mathrm{E}}_{\mathrm{i}}\left({\mathrm{A}}_{\mathrm{i}}^0{\mathrm{A}}_{\mathrm{i}}^0\right)}\hfill & \hfill {\mathbf{G}}_{{\mathrm{E}}_{\mathrm{i}}\left({\mathrm{A}}_{\mathrm{i}}{\mathrm{A}}_{\mathrm{i}}^0\right)}\hfill \\ {}\hfill {\mathbf{G}}_{{\mathrm{E}}_{\mathrm{i}}\left({\mathrm{A}}_{\mathrm{i}}^0{\mathrm{A}}_{\mathrm{i}}\right)}\hfill & \hfill {\mathbf{G}}_{{\mathrm{E}}_{\mathrm{i}}\left({\mathrm{A}}_{\mathrm{i}}{\mathrm{A}}_{\mathrm{i}}\right)}\hfill \end{array}\right] $$, the vector of external EBV for all internal and external animals for the i^th^ source of external information is estimated as:

$$ {\widehat{\mathbf{u}}}_{{\mathrm{E}}_{\mathrm{i}}}=\left[\begin{array}{c}\hfill {\widehat{\mathbf{u}}}_{{\mathrm{E}}_{\mathrm{i}}\left({\mathrm{A}}_{\mathrm{i}}^0\right)}\hfill \\ {}\hfill {\widehat{\mathbf{u}}}_{{\mathrm{E}}_{\mathrm{i}}\left({\mathrm{A}}_{\mathrm{i}}\right)}\hfill \end{array}\right]=\left[\begin{array}{c}\hfill {\mathbf{G}}_{{\mathrm{E}}_{\mathrm{i}}\left({\mathrm{A}}_{\mathrm{i}}^0{\mathrm{A}}_{\mathrm{i}}\right)}{\mathbf{G}}_{{\mathrm{E}}_{\mathrm{i}}\left({\mathrm{A}}_{\mathrm{i}}{\mathrm{A}}_{\mathrm{i}}\right)}^{-1}{\widehat{\mathbf{u}}}_{{\mathrm{E}}_{\mathrm{i}}\left({\mathrm{A}}_{\mathrm{i}}\right)}\hfill \\ {}\hfill {\widehat{\mathbf{u}}}_{{\mathrm{E}}_{\mathrm{i}}\left({\mathrm{A}}_{\mathrm{i}}\right)}\hfill \end{array}\right]. $$

A modified set of multi-trait mixed model equations that integrate *N* sources of external information, each summarized by ${\widehat{\mathbf{u}}}_{{\mathrm{E}}_{\mathrm{i}}}$ and its associated prediction error (co)variance matrix ${\mathbf{D}}_{{\mathrm{E}}_{\mathrm{i}}}$ for the i^th^ source of external information, can be written as [See Additional file 1 for the derivation of the equations]:

1 $$ \begin{array}{l}\left[\begin{array}{cc}\hfill {\mathbf{X}}_{{\mathrm{E}}_0}^{\mathbf{\prime}}{\mathbf{R}}_{{\mathrm{E}}_0}^{-1}{\mathbf{X}}_{{\mathrm{E}}_0}\hfill & \hfill {\mathbf{X}}_{{\mathrm{E}}_0}^{\mathbf{\prime}}{\mathbf{R}}_{{\mathrm{E}}_0}^{-1}{\mathbf{Z}}_{{\mathrm{E}}_0}\hfill \\ {}\hfill {\mathbf{Z}}_{{\mathrm{E}}_0}^{\mathbf{\prime}}{\mathbf{R}}_{{\mathrm{E}}_0}^{-1}{\mathbf{X}}_{{\mathrm{E}}_0}\hfill & \hfill {\mathbf{Z}}_{{\mathrm{E}}_0}^{\mathbf{\prime}}{\mathbf{R}}_{{\mathrm{E}}_0}^{-1}{\mathbf{Z}}_{{\mathrm{E}}_0}+{\mathbf{G}}_{{\mathrm{E}}_0}^{-1}+{\displaystyle \sum_{\mathrm{i}=1}^{\mathrm{N}}\left({\mathbf{D}}_{{\mathrm{E}}_{\mathrm{i}}}^{-1}-{\mathbf{G}}_{{\mathrm{E}}_{\mathrm{i}}}^{-1}\right)}\hfill \end{array}\right]\left[\begin{array}{c}\hfill {\widehat{\boldsymbol{\upbeta}}}_{{\mathrm{E}}_0}\hfill \\ {}\hfill {\widehat{\mathbf{u}}}_{{\mathrm{E}}_0}\hfill \end{array}\right]\\ {}=\left[\begin{array}{c}\hfill {\mathbf{X}}_{{\mathrm{E}}_0}^{\mathbf{\prime}}{\mathbf{R}}_{{\mathrm{E}}_0}^{-1}{\mathbf{y}}_{{\mathrm{E}}_0}\hfill \\ {}\hfill {\mathbf{Z}}_{{\mathrm{E}}_0}^{\mathbf{\prime}}{\mathbf{R}}_{{\mathrm{E}}_0}^{-1}{\mathbf{y}}_{{\mathrm{E}}_0}+{\displaystyle \sum_{\mathrm{i}=1}^{\mathrm{N}}\left({\mathbf{D}}_{{\mathrm{E}}_{\mathrm{i}}}^{-1}{\widehat{\mathbf{u}}}_{{\mathrm{E}}_{\mathrm{i}}}\right)}\hfill \end{array}\right],\end{array} $$

where ${\mathbf{X}}_{{\mathrm{E}}_0}$ and ${\mathbf{Z}}_{{\mathrm{E}}_0}$ are incidence matrices relating records in ${\mathbf{y}}_{{\mathrm{E}}_0}$ to the vector of fixed effects ${\widehat{\boldsymbol{\upbeta}}}_{{\mathrm{E}}_0}$ and the vector of random additive genetic effects ${\widehat{\mathbf{u}}}_{{\mathrm{E}}_0}$, respectively, ${\mathbf{G}}_{{\mathrm{E}}_0}^{-1}$ is the inverse of the internal additive genetic (co)variance matrix associated with the internal genetic evaluation E_0_ that includes all internal and external animals and ${\mathbf{R}}_{{\mathrm{E}}_0}^{-1}$ is the inverse of the residual (co)variance matrix.

For the approximation of ${\mathbf{D}}_{{\mathrm{E}}_{\mathrm{i}}}^{-1}$, it can be shown that [See Additional file 1]: ${\mathbf{D}}_{{\mathrm{E}}_{\mathrm{i}}}^{-1}={\mathbf{G}}_{{\mathrm{E}}_{\mathrm{i}}}^{-1}+{\mathbf{Z}}_{{\mathrm{E}}_{\mathrm{i}}}^{\mathbf{\prime}}{\mathbf{R}}_{{\mathrm{E}}_{\mathrm{i}}}^{-1}{\mathbf{Z}}_{{\mathrm{E}}_{\mathrm{i}}}$, where ${\mathbf{Z}}_{{\mathrm{E}}_{\mathrm{i}}}$ is the incidence matrix relating records of i^th^ external data to internal and external animals and ${\mathbf{R}}_{{\mathrm{E}}_{\mathrm{i}}}^{-1}$ is the residual (co)variance matrix for the i^th^ source of external information. Thereby, ${\mathbf{D}}_{{\mathrm{E}}_{\mathrm{i}}}^{-1}$ is approximated by ${\mathbf{D}}_{{\mathrm{E}}_{\mathrm{i}}}^{-1}={\mathbf{G}}_{{\mathrm{E}}_{\mathrm{i}}}^{-1}+{\boldsymbol{\Lambda}}_{{\mathrm{E}}_{\mathrm{i}}}$, where ${\boldsymbol{\Lambda}}_{{\mathrm{E}}_{\mathrm{i}}}$ is a block diagonal variance matrix with one block per animal [12,14] and ${\boldsymbol{\Lambda}}_{{\mathrm{E}}_{\mathrm{i}}}\approx {\mathbf{Z}}_{{\mathrm{E}}_{\mathrm{i}}}^{\mathbf{\prime}}{\mathbf{R}}_{{\mathrm{E}}_{\mathrm{i}}}^{-1}{\mathbf{Z}}_{{\mathrm{E}}_{\mathrm{i}}}$. Each diagonal block of ${\boldsymbol{\Lambda}}_{{\mathrm{E}}_{\mathrm{i}}}$ is equal to ${\boldsymbol{\Delta}}_{{\mathrm{E}}_{\mathrm{i}}\left(\mathrm{j}\right)}{\mathbf{R}}_0^{-1}{\boldsymbol{\Delta}}_{{\mathrm{E}}_{\mathrm{i}}\left(\mathrm{j}\right)}$ for *j* = *1, 2, …, J* animals, where the matrix **R**_0_ is a matrix of residual (co)variance among traits and the j^th^ matrix ${\boldsymbol{\Delta}}_{{\mathrm{E}}_{\mathrm{i}}\left(\mathrm{j}\right)}$ is a diagonal matrix with elements $\sqrt{{\mathrm{RE}}_{\mathrm{ijk}}}$ where *k* = *1,2,…, K* traits. Element RE_ijk_ is the effective number of records, i.e. record equivalents, for the j^th^ animal for the k^th^ trait associated with the i^th^ source [14,15]. Record equivalents express the quantity of contributions due to relationships and/or due to records considered for the evaluation of an animal. For internal animals, RE_ijk_ is equal to 0 because all contributions are only due to the relationships among external and internal animals. For external animals, if double-counting of contributions due to relationships among them is not taken into account, ${\mathrm{RE}}_{\mathrm{ijk}}=\frac{1-{\mathrm{h}}_{\mathrm{k}}^2}{{\mathrm{h}}_{\mathrm{k}}^2}\ast \frac{{\mathrm{RE}\mathrm{L}}_{\mathrm{ijk}}}{1-{\mathrm{RE}\mathrm{L}}_{\mathrm{ijk}}}$ for the j^th^ animal for the k^th^ trait associated with the i^th^ source, where ${\mathrm{h}}_{\mathrm{k}}^2$ is the heritability of the k^th^ trait [15,18]. If double-counting of contributions due to relationships among external animals is taken into account, RE_ijk_ only expresses the amount of contributions due to records and can be estimated through a two-step algorithm (TSA) [14]. The first step of this TSA determines external animals associated with external information that includes only contributions due to relationships. The second step estimates the amount of contributions due to records (expressed as RE) for external animals associated with information that combines both contributions due to relationships and own records. Note that the proposed approximation of ${\mathbf{Z}}_{{\mathrm{E}}_{\mathrm{i}}}^{\mathbf{\prime}}{\mathbf{R}}_{{\mathrm{E}}_{\mathrm{i}}}^{-1}{\mathbf{Z}}_{{\mathrm{E}}_{\mathrm{i}}}$ differs from the approximation proposed by Quaas and Zhang [12]. Indeed, they proposed to approximate each diagonal block of ${\boldsymbol{\Lambda}}_{{\mathrm{E}}_{\mathrm{i}}}$ by ${\boldsymbol{\Delta}}_{\mathrm{Qi}\left(\mathrm{j}\right)}{\mathbf{G}}_0^{-1}{\boldsymbol{\Delta}}_{\mathrm{Qi}\left(\mathrm{j}\right)}$, where the matrix **G**_0_ is a matrix of genetic (co)variance among traits and **Δ**_Qi(j)_ is a diagonal matrix with elements:

$$ \sqrt{\updelta_{\mathrm{ijk}}}=\sqrt{{\mathrm{REL}}_{\mathrm{ijk}}/\left(1-{\mathrm{REL}}_{\mathrm{ijk}}\right)}. $$

Also, the multi-trait MME (1) that integrate *N* sources of external information differ from the usual multi-trait MME only by the terms ${\displaystyle \sum_{\mathrm{i}=1}^{\mathrm{N}}\left({\mathbf{D}}_{{\mathrm{E}}_{\mathrm{i}}}^{-1}-{\mathbf{G}}_{{\mathrm{E}}_{\mathrm{i}}}^{-1}\right)}$ and ${\displaystyle \sum_{\mathrm{i}=1}^{\mathrm{N}}\left({\mathbf{D}}_{{\mathrm{E}}_{\mathrm{i}}}^{-1}{\widehat{\mathbf{u}}}_{{\mathrm{E}}_{\mathrm{i}}}\right)}$:

2 $$ \begin{array}{l}\left[\begin{array}{cc}\hfill {\mathbf{X}}_{{\mathrm{E}}_0}^{\mathbf{\prime}}{\mathbf{R}}_{{\mathrm{E}}_0}^{-1}{\mathbf{X}}_{{\mathrm{E}}_0}\hfill & \hfill {\mathbf{X}}_{{\mathrm{E}}_0}^{\mathbf{\prime}}{\mathbf{R}}_{{\mathrm{E}}_0}^{-1}{\mathbf{Z}}_{{\mathrm{E}}_0}\hfill \\ {}\hfill {\mathbf{Z}}_{{\mathrm{E}}_0}^{\mathbf{\prime}}{\mathbf{R}}_{{\mathrm{E}}_0}^{-1}{\mathbf{X}}_{{\mathrm{E}}_0}\hfill & \hfill {\mathbf{Z}}_{{\mathrm{E}}_0}^{\mathbf{\prime}}{\mathbf{R}}_{{\mathrm{E}}_0}^{-1}{\mathbf{Z}}_{{\mathrm{E}}_0}+{\mathbf{G}}_{{\mathrm{E}}_0}^{-1}\hfill \end{array}\right]\left[\begin{array}{c}\hfill {\widehat{\boldsymbol{\upbeta}}}_{{\mathrm{E}}_0}\hfill \\ {}\hfill {\widehat{\mathbf{u}}}_{{\mathrm{E}}_0}\hfill \end{array}\right]\\ {}=\left[\begin{array}{c}\hfill {\mathbf{X}}_{{\mathrm{E}}_0}^{\mathbf{\prime}}{\mathbf{R}}_{{\mathrm{E}}_0}^{-1}{\mathbf{y}}_{{\mathrm{E}}_0}\hfill \\ {}\hfill {\mathbf{Z}}_{{\mathrm{E}}_0}^{\mathbf{\prime}}{\mathbf{R}}_{{\mathrm{E}}_0}^{-1}{\mathbf{y}}_{{\mathrm{E}}_0}\hfill \end{array}\right].\end{array} $$

Furthermore, it was previously assumed that the whole pedigree is available for all genetic evaluations. The additive genetic (co)variance matrices that include all internal and external animals are then equal for all genetic evaluations (i.e., ${\mathbf{G}}_{{\mathrm{E}}_0}={\mathbf{G}}_{{\mathrm{E}}_1}={\mathbf{G}}_{{\mathrm{E}}_2}=\dots ={\mathbf{G}}_{{\mathrm{E}}_{\mathrm{N}}}$). Nevertheless, each internal or external genetic evaluation could be performed as a single-step genomic evaluation (e.g., [3,4]) without modifications to the Bayesian derivation [See Additional file 1] because assumptions on the different matrices ${\mathbf{G}}_{{\mathrm{E}}_{\mathrm{i}}}$ were not limiting. Such cases would lead to ${\mathbf{G}}_{{\mathrm{E}}_0}\ne {\mathbf{G}}_{{\mathrm{E}}_{\mathrm{i}}}$. For example, integration of external information provided by the usual MME into a single-step genomic evaluation would lead to ${\mathbf{G}}_{{\mathrm{E}}_0}\ne {\mathbf{G}}_{{\mathrm{E}}_{\mathrm{i}}}$ because ${\mathbf{G}}_{{\mathrm{E}}_0}$ would include genomic information [3,4], unlike ${\mathbf{G}}_{{\mathrm{E}}_{\mathrm{i}}}$.

### Integration of several sources of external information by avoiding double-counting of contributions due to records

Assumptions stated in the previous section led to each source of external information to be obtained from an external evaluation that was based only on external data and free of internal data and information, as well as of the *N*-1 other external data and information. In practice, this assumption is not necessarily valid because a source of external information may be obtained from an external evaluation based on external data and/or information and also on internal data and/or information (e.g., EBV and associated REL obtained in country E_1_ based on external data E_1_ and on internal data E_0_). Thus, double-counting of contributions due to records between internal and external information must be taken into account, as detailed below.

For the i^th^ source of external information, internal information included into external information (subscript I_i_) associated with the external animals can be summarized as ${\widehat{\mathbf{u}}}_{{\mathrm{I}}_{\mathrm{i}}\left({\mathrm{A}}_{\mathrm{i}}\right)}$, i.e. the vector of internal EBV associated with external animals for which external information included both external and internal information, and by ${\mathbf{D}}_{{\mathrm{I}}_{\mathrm{i}}\left({\mathrm{A}}_{\mathrm{i}}\right)}$, the prediction error (co)variance matrix associated with ${\widehat{\mathbf{u}}}_{{\mathrm{I}}_{\mathrm{i}}\left({\mathrm{A}}_{\mathrm{i}}\right)}$.

A modified set of multi-trait mixed model equations that integrate several sources of external information and take double-counting of contributions due to records between external and internal information into account, can be written as follows [See Additional file 2]:

3 $$ \begin{array}{l}\left[\begin{array}{cc}\hfill {\mathbf{X}}_{{\mathrm{E}}_0}^{\mathbf{\prime}}{\mathbf{R}}_{{\mathrm{E}}_0}^{-1}{\mathbf{X}}_{{\mathrm{E}}_0}\hfill & \hfill {\mathbf{X}}_{{\mathrm{E}}_0}^{\mathbf{\prime}}{\mathbf{R}}_{{\mathrm{E}}_0}^{-1}{\mathbf{Z}}_{{\mathrm{E}}_0}\hfill \\ {}\hfill {\mathbf{Z}}_{{\mathrm{E}}_0}^{\mathbf{\prime}}{\mathbf{R}}_{{\mathrm{E}}_0}^{-1}{\mathbf{X}}_{{\mathrm{E}}_0}\hfill & \hfill \begin{array}{l}{\mathbf{Z}}_{{\mathrm{E}}_0}^{\mathbf{\prime}}{\mathbf{R}}_{{\mathrm{E}}_0}^{-1}{\mathbf{Z}}_{{\mathrm{E}}_0}+{\mathbf{G}}_{{\mathrm{E}}_0}^{-1}+\\ {}{\displaystyle \sum_{\mathrm{i}=1}^{\mathrm{N}}\left({\mathbf{D}}_{{\mathrm{E}}_{\mathrm{i}}}^{-1}-{\mathbf{G}}_{{\mathrm{E}}_{\mathrm{i}}}^{-1}\right)}-{\displaystyle \sum_{\mathrm{i}=1}^{\mathrm{N}}\left({\mathbf{D}}_{{\mathrm{I}}_{\mathrm{i}}}^{-1}-{\mathbf{G}}_{{\mathrm{I}}_{\mathrm{i}}}^{-1}\right)}\end{array}\hfill \end{array}\right]\\ {}\left[\begin{array}{c}\hfill {\widehat{\boldsymbol{\upbeta}}}_{{\mathrm{E}}_0}\hfill \\ {}\hfill {\widehat{\mathbf{u}}}_{{\mathrm{E}}_0}\hfill \end{array}\right]=\left[\begin{array}{c}\hfill {\mathbf{X}}_{{\mathrm{E}}_0}^{\mathbf{\prime}}{\mathbf{R}}_{{\mathrm{E}}_0}^{-1}{\mathbf{y}}_{{\mathrm{E}}_0}\hfill \\ {}\hfill {\mathbf{Z}}_{{\mathrm{E}}_0}^{\mathbf{\prime}}{\mathbf{R}}_{{\mathrm{E}}_0}^{-1}{\mathbf{y}}_{{\mathrm{E}}_0}+{\displaystyle \sum_{\mathrm{i}=1}^{\mathrm{N}}\left({\mathbf{D}}_{{\mathrm{E}}_{\mathrm{i}}}^{-1}{\widehat{\mathbf{u}}}_{{\mathrm{E}}_{\mathrm{i}}}\right)}-{\displaystyle \sum_{\mathrm{i}=1}^{\mathrm{N}}\left({\mathbf{D}}_{{\mathrm{I}}_{\mathrm{i}}}^{-1}{\widehat{\mathbf{u}}}_{{\mathrm{I}}_{\mathrm{i}}}\right)}\hfill \end{array}\right],\end{array} $$

where ${\mathbf{G}}_{{\mathrm{I}}_{\mathrm{i}}}$ is a genetic (co)variance matrix for all animals for the internal information included into the i^th^ source of external information, $$ {\widehat{\mathbf{u}}}_{{\mathrm{I}}_{\mathrm{i}}}=\left[\begin{array}{c}\hfill {\widehat{\mathbf{u}}}_{{\mathrm{I}}_{\mathrm{i}}\left({\mathrm{A}}_{\mathrm{i}}^0\right)}\hfill \\ {}\hfill {\widehat{\mathbf{u}}}_{{\mathrm{I}}_{\mathrm{i}}\left({\mathrm{A}}_{\mathrm{i}}\right)}\hfill \end{array}\right]=\left[\begin{array}{c}\hfill {\mathbf{G}}_{{\mathrm{I}}_{\mathrm{i}}\left({\mathrm{A}}_{\mathrm{i}}^0{\mathrm{A}}_{\mathrm{i}}\right)}{\mathbf{G}}_{{\mathrm{I}}_{\mathrm{i}}\left({\mathrm{A}}_{\mathrm{i}}{\mathrm{A}}_{\mathrm{i}}\right)}^{-1}{\widehat{\mathbf{u}}}_{{\mathrm{I}}_{\mathrm{i}}\left({\mathrm{A}}_{\mathrm{i}}\right)}\hfill \\ {}\hfill {\widehat{\mathbf{u}}}_{{\mathrm{I}}_{\mathrm{i}}\left({\mathrm{A}}_{\mathrm{i}}\right)}\hfill \end{array}\right] $$ is the vector of internal EBV associated with the i^th^ source of external information that includes internal information and ${\mathbf{D}}_{{\mathrm{I}}_{\mathrm{i}}}^{-1}$ is the inverse of the prediciton error (co)variance matrix associated with ${\widehat{\mathbf{u}}}_{{\mathrm{I}}_{\mathrm{i}}}$ and approximated as detailed in the previous section.

If the i^th^ source of external information does not include internal information for external animals, the vector ${\widehat{\mathbf{u}}}_{{\mathrm{I}}_{\mathrm{i}}}$ is undetermined and the matrix ${\mathbf{D}}_{{\mathrm{I}}_{\mathrm{i}}}^{-1}$ is equal to ${\mathbf{G}}_{{\mathrm{I}}_{\mathrm{i}}}^{-1}$. This leads to the system of equations (1).

### Blending several sources of external information by avoiding double-counting of contributions due to records

Equations to blend several sources of external information by avoiding double-counting of contributions due to records among internal and external data/information can be derived from the system of equations (3) by assuming that ${\mathbf{y}}_{{\mathrm{E}}_0}$ has no records (i.e. that ${\mathbf{y}}_{{\mathrm{E}}_0}$ is an empty vector). Then, the equation can be written as follows:

4 $$ \begin{array}{l}\left({\mathbf{G}}_{{\mathrm{E}}_0}^{-1}+{\displaystyle \sum_{\mathrm{i}=1}^{\mathrm{N}}\left({\mathbf{D}}_{{\mathrm{E}}_{\mathrm{i}}}^{-1}-{\mathbf{G}}_{{\mathrm{E}}_{\mathrm{i}}}^{-1}\right)}-{\displaystyle \sum_{\mathrm{i}=1}^{\mathrm{N}}\left({\mathbf{D}}_{{\mathrm{I}}_{\mathrm{i}}}^{-1}-{\mathbf{G}}_{{\mathrm{I}}_{\mathrm{i}}}^{-1}\right)}\right)\ {\widehat{\mathbf{u}}}_{{\mathrm{E}}_0}=\\ {}{\displaystyle \sum_{\mathrm{i}=1}^{\mathrm{N}}\left({\mathbf{D}}_{{\mathrm{E}}_{\mathrm{i}}}^{-1}{\widehat{\mathbf{u}}}_{{\mathrm{E}}_{\mathrm{i}}}\right)}-{\displaystyle \sum_{\mathrm{i}=1}^{\mathrm{N}}\left({\mathbf{D}}_{{\mathrm{I}}_{\mathrm{i}}}^{-1}{\widehat{\mathbf{u}}}_{{\mathrm{I}}_{\mathrm{i}}}\right)}.\end{array} $$

### Simulated example

The system of equations (3) was tested using data simulated with the software package GNU Octave [19]. The context of the simulation was a country that imports sires from another country to generate the next generation of production animals and potential sires. Populations of the importing country (hereafter called the internal population) and of the exporting country (hereafter called the external population) were assumed to belong to the same breed. Each population included about 1000 animals distributed over five generations and was simulated from 120 female and 30 male founders. For both populations, milk yield in the first lactation was simulated for each female with progeny, following Van Vleck [20]. A herd effect nested within-population was randomly assigned to each phenotypic record. To obtain enough observations per level for the herd effect, each herd included at least 40 females. Phenotypic variance and heritability were assumed to be 3.24*10^6^ kg^2^ and 0.25, respectively.

To simulate the internal and external populations, the following rules were applied to generate each new generation. First, from the second generation, both females and males older than one year old were considered as mature for breeding and a male could be mated during at most two breeding years. Second, 95% of the available females and 75% of the available males with the highest true breeding values were selected for breeding. Third, all selected females were randomly mated with the selected males. The maximum number of males mated to produce the next generation was set to 25. Furthermore, a mating could be performed only if the additive relationship coefficient between male and female was less than 0.5 and if the female had less than three progeny.

The external population was simulated first and additional rules were applied to this population. For this population, males that were selected for mating only originated from the external population and 60% of the external male offspring with the lowest true breeding values were culled in each generation. Then, the internal population was simulated. For this population, males were selected among all available internal males and a subset of selected external sires. This subset of external sires included the first 50 sires with the highest true breeding values in the external population. Also, 99% of internal male offspring with the lowest true breeding values were culled in each generation. No female offspring was culled in either population.

Using the simulated data, three genetic evaluations were performed (Table 2):

- A joint evaluation (EVAL_J_) was performed as a BLUP evaluation using the system of equations (2) and based on external and internal pedigree and data. This evaluation was assumed to be the reference.
- An internal evaluation (EVAL_I_) was performed as a BLUP evaluation using the system of equations (2) and based on internal pedigree and data.
- An external evaluation (EVAL_E_) was performed as a BLUP evaluation using the system of equations (2) and based on external pedigree and data.

**Table 2:** **Genetic evaluations performed for the simulated example**

	**Genetic evaluations** ^**1**^					
	**J**	**E**	**I**	**BE**	**BJ**	**BJ-I**
External pedigree	X	X				
Internal pedigree	X		X	X	X	X
External data	X	X				
Internal data	X		X	X	X	X
Integrated information (50 external sires)						
External EBV and REL				X		
Joint EBV and REL					X	X
Internal EBV and REL						X

^1^J = Joint; E = External; I = Internal; BE = Bayesian External; BJ = Bayesian Joint; BJ-I = Bayesian Joint minus Internal.

Three Bayesian evaluations that integrated information provided by EVAL_E_ or by EVAL_J_ for the 50 external sires into EVAL_I_ were also performed. Because the external sires were related, double-counting of contributions due to relationships existed and this was taken into account for the three Bayesian evaluations through the TSA [14]. Double-counting of contributions due to records could also exist with the integration of information provided by EVAL_J_ into EVAL_I_ because EVAL_J_ and EVAL_I_ were partially based on the same data (i.e., internal data). The following three Bayesian evaluations were performed:

(d) A Bayesian evaluation using the system of equations (1) and using EBV and PEV obtained from EVAL_E_ associated with the 50 external sires that were used inside the internal population as external information (EVAL_BE_).
(e) A Bayesian evaluation using the system of equations (1) and EBV and PEV obtained from EVAL_J_ associated with the 50 external sires as external information (hereafter called joint information) (EVAL_BJ_). Although EVAL_J_ was based on external and internal data, double-counting of contributions due to records between joint and internal information was not taken into account.
(f) A Bayesian evaluation integrating joint information by using the system of equations (3) and taking into account double-counting of contributions due records among internal and joint information (EVAL_BJ-I_). Double-counting of contributions due to records among internal and joint information was taken into account by using EBV and PEV obtained from EVAL_I_ associated with the 50 external sires.

The simulation was replicated 100 times. Comparisons between EVAL_J_ and EVAL_I_, EVAL_BE_, EVAL_BJ_, or EVAL_BJ-I_ were performed separately for the 50 external sires and for the internal animals. Comparisons were based on:

1. Spearman’s rank correlation coefficients (r) of EBV obtained from EVAL_J_ (EBV_J_) with EBV obtained from EVAL_I_ (EBV_I_), EVAL_BE_ (EBV_BE_), EVAL_BJ_ (EBV_BJ_), and EVAL_BJ-I_ (EBV_BJ-I_),
2. regression coefficients (a) of EBV_J_ on EBV_I_, EBV_BE_, EBV_BJ_, and EBV_BJ-I_, and
3. coefficients of determination (R^2^) associated with the regressions,
4. the total amount of RE (RE_tot_) associated with external information, joint information and joint information corrected for the included internal information, and
5. mean squared errors (MSE) of EBV_I_, EBV_BE_, EBV_BJ_, and EBV_BJ-I_, expressed as a percentage of MSE obtained for EBV_I_. For each replicate, the MSE obtained for EBV_I_ was reported to a relative value of 100 before the different computations of MSE.

Because the TSA was applied before all three Bayesian evaluations, RE_tot_ were free of contributions due to relationships estimated by the Bayesian evaluations. For an easier understanding of the results and discussion, RE can be transformed into daughter equivalents (DE) through ${\mathrm{DE}}_{\mathrm{ijk}}=\frac{4-{\mathrm{h}}_{\mathrm{k}}^2}{1-{\mathrm{h}}_{\mathrm{k}}^2}\ast {\mathrm{RE}}_{\mathrm{ijk}}$ [18]. All results were the average of the 100 replicates.

### Walloon example

Even if MACE allows the aggregation of EBV for dairy sires, internal genetic evaluations for animals not associated with MACE information (e.g., cows, calves, young sires) are not influenced by external information considered by the MACE for dairy sires and may be still biased. Therefore, integration of MACE information into internal evaluations, as well as blending of MACE and internal information, could benefit those animals. The performance of equation (4) that blends MACE and internal information was evaluated in the context of the official Walloon genetic evaluation for Holstein cattle.

The Walloon example used information for milk, fat and protein yields for Holstein cattle provided by the official Walloon genetic evaluation [21,22]. The genetic variances were those used for the official Walloon genetic evaluation [21] and were equal to 280 425 kg^2^ for milk yield, to 522.6 kg^2^ for fat yield and to 261.5 kg^2^ for protein yield. The respective heritabilities were equal to 0.38, 0.43 and 0.41. The pedigree file was extracted from the database used for the official Walloon genetic evaluation (EVAL_W_) and covered up to six known ancestral generations. The extraction was performed for a randomly selected group of 1909 animals (potentially genotyped) born after 1998. The selected group included sires, cows and calves that were used or were not at the internal level. After extraction, the pedigree file contained 16 234 animals.

Internal information included EBV and associated REL estimated from data provided by the Walloon Breeding Association (EBV_W_, REL_W_) for the EVAL_W_ for milk production of April 2013 [21,22]. A total of 12 046 animals were associated with an available EBV_W_. External information included EBV and REL for 1981 sires provided with the official release for the April 2013 MACE performed by Interbull (EVAL_MACE_, EBV_MACE_, REL_MACE_) [23]. It should be noted that the Walloon region in Belgium participated in the April 2013 MACE. Internal and external information were harmonized between the Walloon and MACE evaluations by adjusting scales and mean differences towards the original expression of the trait in the Walloon genetic evaluations. External information was then considered to be the same trait as the internal phenotype trait.

Unlike the simulated example, no joint evaluation based on Walloon and external records was available for both external and internal animals. Because EVAL_MACE_ aggregated EBV from several national genetic evaluations for sires, it was considered as the reference for the evaluated sires. Walloon and MACE information were blended by using equation (4) for the following four cases: with or without consideration of double-counting of contributions due to relationships and with or without consideration of double-counting of contributions due to records (Table 3). Double-counting of contributions due to relationships was possible because all animals associated with Walloon and/or MACE information were related. Double-counting of contributions due to records was also possible because MACE information associated with the 1981 sires included contributions provided by EVAL_W_. Thus, to test the importance of both double-counting issues, the following four cases were evaluated:

- Walloon and MACE information were blended without considering double-counting of contributions due to records and due to relationships (EVAL_BLNN_, EBV_BLNN_, REL_BLNN_).
- Walloon and MACE information were blended by considering only double-counting of contributions due to records (EVAL_BLRE_, EBV_BLRE_, REL_BLRE_). To achieve this goal, the contribution of Walloon information into MACE information was determined based on the domestic effective daughter equivalents (EDC) associated with EBV_MACE_ and REL_MACE_ and provided with the official release for the 2013 April MACE by Interbull. MACE information free of Walloon information was reported by a domestic EDC equal to 0. A total of 601 sires were associated with an EDC greater than 0. For these 601 sires, EBV and associated REL estimated from Walloon data and contributing to the April 2013 MACE routine-run (EBV_Wc_, REL_Wc_) were considered by EVAL_BLRE_ to take double-counting of contributions due to records into account. Double-counting of contributions due to relationships was not taken into account for either Walloon or MACE information.
- Walloon and MACE information were blended by only considering double-counting of contributions due to relationships among all animals (EVAL_BLR_, EBV_BLR_, REL_BLR_). The TSA was therefore applied for Walloon and MACE information. Double-counting of contributions due to records was not considered.
- Walloon and MACE information were blended by considering both double-counting of contributions due to records and due to relationships (EVAL_BL_, EBV_BL_, REL_BL_). Reliabilities for EBV_BLNN_, EBV_BLRE_, EBV_BLR_ and EBV_BL_ were computed using the equation $REL=1-PEV/{\sigma}_g^2$, where ${\sigma}_g^2$ is the genetic variance for the corresponding trait and *PEV* is the prediction error variance obtained from the diagonal element of the inverted left-hand-side of the equation (4).

**Table 3:** **Bayesian evaluations performed for the Walloon example**

	**Bayesian evaluations**			
	**BLNN**	**BLRE**	**BLR**	**BL**
Available estimated breeding values and reliabilities				
Official Walloon evaluation	X	X	X	X
Multiple Across Country Evaluation	X	X	X	X
Double-counting accounted				
Records		X		X
Relationships			X	X

As explained previously, EVAL_MACE_ was considered as the reference for sires evaluated through EVAL_MACE_. Comparisons between EVAL_MACE_ and EVAL_W_, EVAL_BLNN_, EVAL_BLRE_, EVAL_BLR_ or EVAL_BL_ were performed based on:

1. Spearman’s rank correlation coefficients (r) of EBV_MACE_ with EBV_W_, EBV_BLNN_, EBV_BLRE_, EBV_BLR_ and EBV_BL_,
2. MSE of EBV_W_, EBV_BLNN_, EBV_BLRE_, EBV_BLR_, and EVAL_BL_ (i.e. mean squared errors expressed as a percentage of average MSE of EBV_W_),
3. regression coefficients (a) and,
4. R^2^ of the regressions of EVAL_MACE_ on the five other evaluations (i.e., EVAL_W_, EVAL_BLNN_, EVAL_BLRE_, EVAL_BLR_ and EVAL_BL_),
5. RE_tot_ and (6) average REL.

Comparisons concerned two groups of sires. A first group of sires included 1212 sires that were associated with both Walloon and MACE information and had daughters with records in the Walloon region dataset (hereafter called “internally used sires”). A second group of sires included 631 sires that were associated with both Walloon and MACE information but had no daughters with records in the Walloon region dataset (i.e. they had only foreign, or external, daughters; hereafter called “internally unused sires”). The RE_tot_ were free of contributions due to relationships that were estimated by the Bayesian evaluations but could include contributions due to relationships that resulted from the previous genetic evaluation if the TSA was not applied.

The effect of blending MACE and Walloon information was also studied for internal animals that were not associated with MACE information and that were sired by internally used sires by considering (1) r between EVAL_BL_ and EVAL_W_, EVAL_BLNN_, EVAL_BLRE_ or EVAL_BLR_, (2) RE_tot_ and (3) average REL. Three groups of internal animals were defined depending on their REL_W_. The first group included internal animals that were associated with a REL_W_ lower than 0.50, the second group included internal animals that were associated with a REL_W_ between 0.50 and 0.75, and the third group included internal animals with a REL_W_ equal or higher than 0.75.

All blending evaluations were performed using a version of the BLUPF90 program [24] modified to implement the equations (1), (3) and (4).

## Results and discussion

### Simulated example

On average, each of the 100 simulated internal and external populations included 1048 animals. Results for r, MSE, a and R^2^ for prediction of EBV_J_ are in Table 4 for the 50 external sires and for the internal animals.

**Table 4:** **Average (SD in parentheses) of parameters obtained for the simulated example over 100 replicates**

**Concerned animals** ^**1**^	**Genetic evaluation** ^**2**^	**r** ^**2**^	**MSE** ^**3**^	**a** ^**4**^	**R** ^**2 4**^	**RE**_**tot**_^**5**^
Internal animals						
	EVAL_I_	0.934 (0.021)	100.00 (28.621)	0.982 (0.042)	0.896 (0.030)	-
	EVAL_BE_	>0.999 (0.000)	0.61 (0.58)	0.997 (0.005)	0.999 (0.001)	-
	EVAL_BJ_	0.979 (0.005)	34.26 (7.92)	0.977 (0.024)	0.965 (0.008)	-
	EVAL_BJ-I_	0.996 (0.001)	6.78 (3.02)	1.021 (0.013)	0.993 (0.002)	-
External sires						
	EVAL_I_	0.571 (0.131)	100.00 (32.31)	0.712 (0.168)	0.391 (0.146)	-
	EVAL_BE_	0.997 (0.001)	0.35 (0.22)	1.000 (0.011)	0.998 (0.002)	76.3 (5.1)
	EVAL_BJ_	0.956 (0.017)	17.16 (4.18)	0.821 (0.039)	0.924 (0.030)	141.5 (7.8)
	EVAL_BJ-I_	0.996 (0.002)	0.60 (0.26)	0.993 (0.012)	0.996 (0.002)	78.7 (5.1)

^1^Internal animals = animals associated with only internal information; External sires: sires associated with external information; ^2^EVAL_I_ = BLUP evaluation based on internal pedigree and data; EVAL_BE_ = Bayesian evaluation using external EBV and PEV associated with the 50 external sires used in the internal population; EVAL_BJ_ = Bayesian evaluation using EBV and PEV obtained from the joint evaluation and associated with the 50 external sires; EVAL_BJ-I_ = Bayesian evaluation using EBV and PEV obtained from the joint and from internal evaluations and associated with the 50 external sires to avoid double-counting among internal and joint information; r^2^ = rank correlations between EBV estimated by EVAL_J_ and by EVAL_I_, EVAL_BE_, EVAL_BJ_ or EVAL_BJ-I;_^3^MSE = mean squared errors expressed as a percentage of the average internal MSE between a joint evaluation and EVAL_I_, EVAL_BE_, EVAL_BJ_ or EVAL_BJ-I_; ^4^a = regression coefficient and R^2^ = coefficient of determination of the regression of EBV estimated by the joint evaluation on EBV estimated by EVAL_I_, EVAL_BE_, EVAL_BJ_ or EVAL_BJ-I_; ^5^RE_tot_ = total amount of record equivalents free of contributions due to relationships among external animals.

Compared to the rankings of EVAL_I_, integration of external or joint information for the 50 external sires led to rankings of EVAL_BE_, EVAL_BJ_ or EVAL_BJ-I_ that were more similar to those of EVAL_J_. Rank correlations r increased from 0.57 for EVAL_I_ to at least 0.95 for EVAL_BJ_ for the 50 external sires and from 0.93 for EVAL_I_ to at least 0.98 for EVAL_BJ_ for internal animals (Table 4). Furthermore, MSE, a and R^2^ also showed that the integration of external or joint information for the 50 external animals with EVAL_BE_, EVAL_BJ_ or EVAL_BJ-I_ led to better predictions of EBV_J_ for both external and internal animals (Table 4). Therefore, the observations that internals animals related to the 50 external sires were also better predicted by EVAL_BE_, EVAL_BJ_ and EVAL_BJ-I_, compared to EVAL_I_, revealed that the external information propagated from the 50 external sires to relatives.

The RE_tot_ associated with EVAL_BE_ was equal to 76.3 (which also corresponded to 381.6 DE), while the RE_tot_ associated with EVAL_BJ_ was equal to 141.5 (DE = 707.7, Table 4). The higher RE_tot_ associated with EVAL_BJ_ showed that double-counting of contributions due to records was present when joint information was integrated. Indeed, joint information contained both external and internal information. The RE_tot_ associated with EVAL_BJ-I_ was equal to 78.7 (DE = 393.3, Table 4). While this latter RE_tot_ is slightly higher (i.e. 3.1% on average) than the RE_tot_ associated with EVAL_BE_, it showed that double-counting was almost avoided when internal information was considered for the 50 external sires. A total of 96.4% of contributions due to records of internal information on average was removed from the joint information (Table 4). The remaining 3.6% of contributions due to records of internal information was double-counted by the Bayesian evaluations and may result from the estimation of contributions due to relationships and/or from the estimation of contributions due to records among joint and internal information.

Because double-counting of contributions due to records between joint and internal information was almost avoided, breeding values that were estimated by EVAL_BJ-I_ for all animals led to better predictions of EBV_J_ than EVAL_BJ_, based on r, MSE, a and R^2^ (Table 4). Rank correlations of EBV_J_ with EBV_BJ_ and EBV_BJ-I_ increased from 0.979 for EVAL_BJ_ to 0.996 for EVAL_BJ-I_ for the internal animals and from 0.956 for EVAL_BJ_ to 0.996 for EVAL_BJ-I_ for the 50 external animals. The MSE decreased on average from 34.3% for EVAL_BJ_ to 6.8% for EVAL_BJ-I_ for the internal animals and from 17.2% for EVAL_BJ_ to 0.6% for EVAL_BJ-I_ for the external animals. These results again showed that integration of external/joint information for the 50 external sires influenced the prediction of internal relatives through the propagation of information from the external sires to relatives. These results show that the double-counting of contributions due to records also affected predictions of internal animals. Furthermore, as expected, EVAL_BE_ predicted EBV_J_ slightly better than EVAL_BJ-I_ for both external sires and internal animals, based on the corresponding r, MSE, a and R^2^ (Table 4). The low difference in accuracy of prediction between EVAL_BE_ and EVAL_BJ-I_ could be attributed to the estimation of contributions due to relationships and due to records.

Based on these results, double-counting of contributions due to records was almost avoided. Thus, the integration of information into a genetic evaluation by avoiding both contributions due to relationships and due to records performed well for external animals. Internal animals also benefited of the integration of information thanks to their relationships with external animals.

### Walloon example

**Figure 1:**
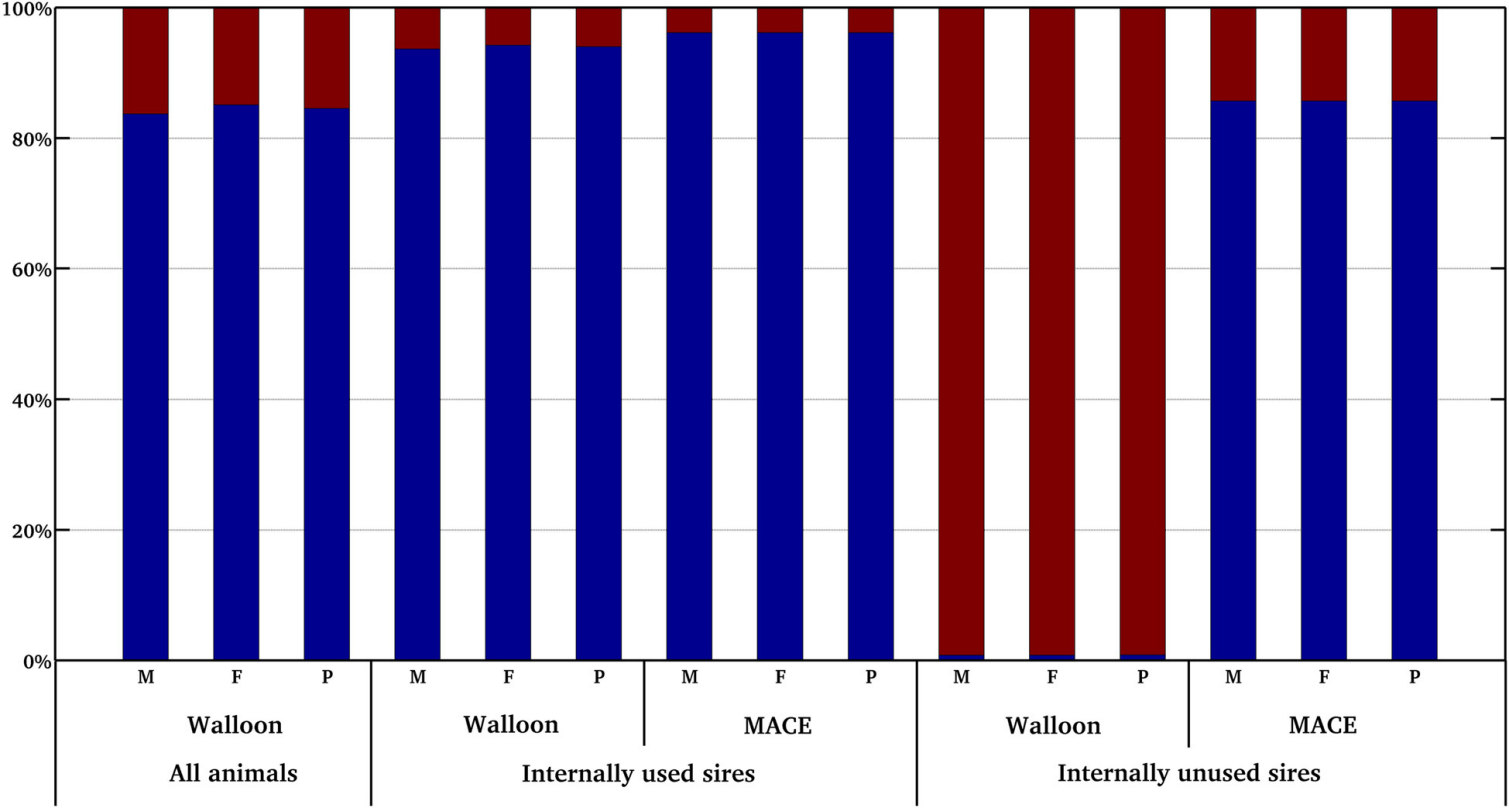
**Percentage of contributions due to records and due to relationships for the Walloon example.** Percentage of contributions due to records (blue squares) and due to relationships (red squares) associated with Walloon information for all animals, internally used and unused sires and associated with MACE information for internally used and unused sires for milk (M), fat yield (F) and protein (P) yields.

Of the 12 046 animals associated with available Walloon information for the three traits, 6232 animals for milk yield, 6209 animals for fat yield, and 6212 animals for protein yield were associated with information that was based only on contributions due to relationships, as estimated by the TSA. In terms of RE, contributions due to relationships represented from 14.9% for fat yield to 16.3% for milk yield of the contributions associated with Walloon information (Figure 1). Among the 1981 sires associated with MACE information, two sires were associated with information that includes only contributions due to relationships for the three traits. Both these sires had several sons among all the sires associated with an EBV_MACE_, which explains that the contributions were considered as only due to relationships. In terms of RE, all contributions due to relationships represented on average 5.1% of the contributions associated with MACE information for the three traits. Of the 601 sires with an EBV_Wc_, all sires were associated with information that included both contributions due to relationships and due to records. This latter observation for the 601 sires was expected because these 601 sires must have at least 10 daughters with records within 10 herds in the Walloon region to participate in the MACE evaluation.

#### Internally used sires

Of the internally used sires, 1212 had Walloon and MACE information and had both internal and external daughters with records. On average, each sire had 143.1 internal daughters with records. The average REL_W_ ranged from 0.74 to 0.76 (Table 5) and the average REL_MACE_ was equal to 0.88 for the three traits. Results for r, MSE, a and R^2^ for prediction of EBV_MACE_ by EVAL_BL_ are in Table 6 for the 1212 sires for milk, fat and protein yields. For the three traits, blending of Walloon and MACE information by taking double-counting of contributions due to records and due to relationships into account (i.e. EVAL_BL_) led to a ranking that was more similar to the MACE ranking than to the internal ranking (i.e. EVAL_W_), although these internally used sires sired a large number of cows with records in the Walloon region. Rank correlations increased by 0.104 points for milk yield to 0.125 points for fat yield to achieve a rank correlation between EBV_MACE_ and EBV_BL_ that ranged from 0.987 to 0.990 (Table 6). The MSE, a and R^2^ showed that accuracy of predictions of EBV_MACE_ by EBV_W_ or by EBV_BL_ increased when external information was integrated. Integration of MACE information also increased the average REL by 0.14 points for fat yield to 0.16 points for milk yield (Table 5). This increase of average REL corresponded to an increase of 57.5, 51.4, and 50.9 DE per sire on average for milk, fat and protein yields, respectively. Also, the average REL_BL_ for the 1212 sires was 0.02 points higher than the average REL_MACE_ (Table 6). This difference in average REL, as well as the differences between EBV_MACE_ and EBV_BL_ based on MSE, a and R^2^ (Table 6), can be explained by the fact that MACE did not include all information available for animals in the Walloon region. Indeed, EBV_W_ of a sire was included into MACE if it had at least10 daughters with records within 10 herds at the internal level. Therefore, EBV_W_ for sires that did not fulfill this requirement were not considered by MACE, but were taken into account by the four Bayesian evaluations, which provided additional information compared to MACE information. Approximations based on estimation of contributions due to relationships and theoretical assumptions of the model may also explain some of the differences between EBV_MACE_ and EBV_BL_. For example, MACE was considered as a national genetic evaluation. These results indicate that EVAL_BL_, i.e. a Bayesian evaluation that blended internal information and external information and avoided most double-counting of contributions due to records and due to relationships, was successful in integrating MACE information for internally used sires.

**Table 5:** **Average reliabilities (REL; SD in parentheses) associated with Walloon estimated breeding values for internally used and unused sires**

**Considered animals**	**Milk yield**	**Fat yield**	**Protein yield**
Internally used sires	0.74 (0.22)	0.76 (0.21)	0.75 (0.22)
Internally unused sires	0.22 (0.10)	0.23 (0.10)	0.22 (0.10)

**Table 6:** **Parameters obtained for the Walloon example for 1212 internally used sires**

**Genetic evaluations**	**Milk yield**					
	**r** ^**1**^	**MSE** ^**2**^	**a** ^**3**^	**R** ^**2 3**^	**RE**_**tot**_^**4**^	**REL** ^**5**^
EVAL_W_	0.886	100.00	0.87 (0.013)	0.78	21 934.6	0.74 (0.22)
EVAL_BLNN_	0.987	11.68	0.993 (0.005)	0.97	55 038.2	0.92 (0.05)
EVAL_BLRE_	0.989	10.01	0.984 (0.004)	0.98	37 487.1	0.91 (0.05)
EVAL_BLR_	0.988	10.57	1.004 (0.004)	0.98	52 313.0	0.91 (0.06)
EVAL_BL_	0.990	8.87	0.995 (0.004)	0.98	34 141.2	0.90 (0.06)
	**Fat yield**					
	**r** ^**1**^	**MSE** ^**2**^	**a** ^**3**^	**R** ^**2 3**^	**RE**_**tot**_^**4**^	**REL** ^**5**^
EVAL_W_	0.862	100.00	0.815 (0.014)	0.74	20 016.8	0.76 (0.22)
EVAL_BLNN_	0.983	12.22	0.989 (0.005)	0.97	46 144.6	0.92 (0.05)
EVAL_BLRE_	0.985	10.69	0.977 (0.005)	0.97	32 320.9	0.92 (0.05)
EVAL_BLR_	0.985	11.12	1.004 (0.005)	0.97	43 943.6	0.91 (0.06)
EVAL_BL_	0.987	9.54	0.991 (0.005)	0.97	29 631.1	0.90 (0.06)
	**Protein yield**					
	**r** ^**1**^	**MSE** ^**2**^	**a** ^**3**^	**R** ^**2 3**^	**RE**_**tot**_^**4**^	**REL** ^**5**^
EVAL_W_	0.882	100.00	0.851 (0.013)	0.79	20 851.6	0.75 (0.22)
EVAL_BLNN_	0.985	12.38	0.985 (0.005)	0.97	49 589.7	0.92 (0.05)
EVAL_BLRE_	0.987	10.79	0.975 (0.004)	0.98	34 372.9	0.91 (0.05)
EVAL_BLR_	0.986	11.26	0.996 (0.005)	0.98	47 189.5	0.91 (0.06)
EVAL_BL_	0.988	9.56	0.986 (0.004)	0.98	31 434.7	0.90 (0.06)

^1^r = rank correlation between EVAL_MACE_ and EVAL_W_, EVAL_BLNN_, EVAL_BLRE_, EVAL_BLR_ or EVAL_BL_; ^2^MSE = mean squared error expressed as a percentage of the average internal mean squared error; ^3^a = regression coefficient (SE in parentheses) and R^2^ = coefficient of determination of the regression of MACE EBV on EBV estimated by EVAL_W_, EVAL_BLNN_, EVAL_BLRE_, EVAL_BLR_ or EVAL_BL_; ^4^RE_tot_ = total amount of record equivalents; ^5^REL = average reliability (SD in parentheses).

Double-counting of contributions due to records and due to relationships were also not considered (i.e. EVAL_BLNN_) or were considered separately (i.e. EVAL_BLRE_ and EVAL_BLR_) to study their influences on prediction of EVAL_MACE_ for internally used sires. Parameters r, a and R^2^ associated with EVAL_BLNN_, EVAL_BLRE_ and EVAL_BLR_ for the 1212 sires were similar to the r, a and R^2^ of EVAL_BL_, although a slight advantage was observed for EVAL_BL_. Therefore, the four blending evaluations led to similar rankings as MACE for the 1212 internally used sires (i.e., rank correlations equal to 0.99 on average; Table 6).

However, double-counting can be observed based on MSE, RE_tot_ and REL (Table 6). With regard to double-counting of contributions due to relationships for the 1212 internally used sires, RE that were free of contributions due to relationships (i.e. RE that included only contributions due to records) for EBV_MACE_ were equal to 30 378 (DE = 176 578) for milk yield, 23 927 (DE = 150 772) for fat yield, and 26 338 (DE = 160 416) for protein yield. These amounts of RE free of contributions due to relationships represented 96.1% of the RE that contributed to MACE information. Considering the Walloon information for the 1212 sires, RE that included only contributions due to records represented from 93.6% of all Walloon contributions for milk yield to 94.2% for fat yield. For both Walloon and MACE information associated with the internally used sires and for the three traits (i.e. for milk, fat and protein yields), less than 6.4% of all contributions were attributed to relationships (Figure 1). Such low percentages of contributions due to relationships are in agreement with selection index theory [25]. While double-counting of contributions due to relationships was present for EVAL_BLRE_ (i.e. the blending evaluation that considered only double-counting of contributions due to records), the contributions due to relationships were small and their double-counting had little effect on the prediction of EBV_MACE_ for the internally used sires, compared to EVAL_BL_, based on parameters r and MSE. However, as expected, an average increase of 1% in REL_BLRE_ was observed, compared to REL_BL_. Thus, the REL_BLRE_ were, on average, slightly overestimated.

With regard to double-counting of contributions due to records, based on RE, Walloon information represented from 64.3% of the total information free of contributions due to relationships associated with EVAL_BL_ for milk yield to 67.6% for fat yield (Table 6). Thus, integrated information free of contributions due to relationships and due to records (i.e. MACE information from which Walloon information was subtracted) represented 32.5% of the total information associated with EVAL_BL_ for fat yield to 35.8% for milk yield. If double-counting of contributions due to relationships was considered only, RE_tot_ associated with EVAL_BLR_ ranged from 43 944 RE for fat yield to 52 313 RE for milk yield, while RE_tot_ associated with EVAL_BL_ ranged from 29 631 RE for fat yield to 34 141 RE for milk yield. Thus, between 14 313 and 18 172 RE were considered twice by EVAL_BLR_. However, double-counting of contributions due to records affected the prediction of EBV_MACE_ for internally used sires only slightly according to all parameters evaluated (Table 5). The REL_BLR_ were overestimated by 1% on average for the internally used sires, compared to REL_BL_. Furthermore, no preference was observed between EVAL_BLRE_ and EVAL_BLR_ based on r, MSE, a and R^2^ for the three traits. Indeed, r and R^2^ were similar for these two evaluations, while EVAL_BLRE_ was more reliable based on MSE, but parameter a indicated that EVAL_BLR_ was more reliable. However, EVAL_BLRE_ had the greatest under- and overestimation of true breeding values based on parameter a. Based on these results, it can be stated that double-counting of contributions due to relationships and due to records had little effect on EBV for internally used sires.

#### Internally unused sires

Of the internally unused sires (i.e. that had only external daughters with records), 631 sires were associated with Walloon and MACE information. Their average REL_W_ ranged from 0.22 to 0.23 for the three traits (Table 7) and the average REL_MACE_ was equal to 0.77. Because they had only external daughters, Walloon contributions only included contributions due to relationships and no contributions due to records. Based on RE_tot_ (Table 7), Walloon contributions due to records for all 631 sires were in general well estimated by the TSA, ranging from 0.79% of the Walloon total contributions for milk yield to 0.80% for protein yield (Figure 1). The small non-zero percentage could be attributed to approximations involved in estimating the contributions due to relationships and due to records by the TSA, such as the consideration of an unknown fixed effect [14]. The nearly correct estimation of contributions due to relationships led to similar average REL_MACE_ and average REL_BL_ for the three traits (Table 7). Integration of MACE information also increased the average REL_W_ by at least 0.54 points, resulting in an average REL_BL_ equal to 0.77 for the three traits. These results for the 631 internally unused sires confirmed that MACE information already contained the main contributions due to relationships that were expressed in the Walloon information and that double-counting of contributions due to relationships was mostly avoided. Not considering contributions due to relationships (i.e. EVAL_BLNN_ and EVAL_BLRE_) led to overestimation of average REL by at least 3% (Table 7).

**Table 7:** **Parameters obtained for the Walloon example for 631 internally unused sires**

**Genetic evaluations**	**Milk yield**					
	**r** ^**1**^	**MSE** ^**2**^	**a** ^**3**^	**R** ^**2 3**^	**RE**_**tot**_^**4**^	**REL** ^**5**^
EVAL_W_	0.725	100.00	0.667 (0.024)	0.56	2.5	0.22 (0.10)
EVAL_BLNN_	0.994	3.09	0.953 (0.004)	0.99	4021.7	0.81 (0.05)
EVAL_BLRE_	0.994	3.06	0.952 (0.004)	0.99	4021.7	0.81 (0.05)
EVAL_BLR_	0.994	2.68	0.978 (0.004)	0.99	3172.9	0.77 (0.06)
EVAL_BL_	0.994	2.68	0.977 (0.004)	0.99	3172.9	0.77 (0.06)
	**Fat yield**					
	**r** ^**1**^	**MSE** ^**2**^	**a** ^**3**^	**R** ^**2 3**^	**RE**_**tot**_^**4**^	**REL** ^**5**^
EVAL_W_	0.571	100.00	0.506 (0.024)	0.40	2.0	0.23 (0.10)
EVAL_BLNN_	0.992	2.28	0.95 (0.005)	0.99	3172.5	0.81 (0.05)
EVAL_BLRE_	0.992	2.28	0.949 (0.005)	0.99	3172.5	0.81 (0.05)
EVAL_BLR_	0.992	2.09	0.987 (0.005)	0.99	2499.1	0.77 (0.06)
EVAL_BL_	0.992	2.08	0.986 (0.005)	0.99	2499.1	0.77 (0.06)
	**Protein yield**					
	**r** ^**1**^	**MSE** ^**2**^	**a** ^**3**^	**R** ^**2 3**^	**RE**_**tot**_^**4**^	**REL** ^**5**^
EVAL_W_	0.717	100.00	0.684 (0.025)	0.54	2.3	0.22 (0.10)
EVAL_BLNN_	0.993	2.96	0.952 (0.004)	0.99	3490.3	0.81 (0.05)
EVAL_BLRE_	0.993	2.95	0.951 (0.004)	0.99	3490.3	0.81 (0.05)
EVAL_BLR_	0.993	2.75	0.978 (0.005)	0.99	2751.0	0.78 (0.06)
EVAL_BL_	0.993	2.75	0.977 (0.005)	0.99	2751.0	0.77 (0.06)

^1^r = rank correlation between EVAL_MACE_ and EVAL_W_, EVAL_BLNN_, EVAL_BLRE_, EVAL_BLR_ or EVAL_BL_; ^2^MSE = mean squared error expressed as a percentage of the average internal mean squared error; ^3^a = regression coefficient (SE in parentheses) and R^2^ = coefficient of determination of the regression of MACE EBV on EBV estimated by EVAL_W_, EVAL_BLNN_, EVAL_BLRE_, EVAL_BLR_ or EVAL_BL_; ^4^RE_tot_ = total amount of record equivalents; ^5^REL = average reliability (SD in parentheses).

Results for r, MSE, a and R^2^ for the prediction of EBV_MACE_ by the four blending evaluations are in Table 7 for the 631 internally unused sires for the three traits. Blending of Walloon and MACE information led to similar rankings of the 631 sires for the four blending evaluations. Rank correlations between EBV_MACE_ and EBV for the four blending evaluations increased from 0.73 to 0.99 for milk yield, from 0.57 to 0.99 for fat yield and from 0.72 to 0.99 for protein yield. These rank correlations indicated that the blending method was also successful for sires with only external information for all three traits. These results were confirmed by a decrease of MSE by at least 96.9% and by regression coefficients close to 1.0, with an R^2^ equal to 0.99 for all three traits (Table 7). Because double-counting can be only attributed to contributions due to relationships for the 631 internally unused sires, EVAL_BLNN_ and EVAL_BLRE_ led to similar parameters. This was also observed for EVAL_BL_ and EVAL_BLR_ (Table 7). Differences between these two groups of evaluations were only observed based on MSE and a (Table 7). These two parameters showed that EBV_MACE_ for the 631 sires were slightly better predicted when contributions due to relationships were considered. However, all these results showed that contributions due to relationships had little effect on the prediction of EBV_MACE_.

VanRaden and Tooker [17] found similar correlations between EBV_MACE_ and combined EBV for sires with only external daughters (i.e. between 0.991 and 0.994 for yield traits). Their strategy consisted of computing external deregressed proofs (DRP) from EBV_MACE_ and including one extra record based on these DRP, weighted by the associated DE for the sire. Internal contributions in MACE information for sires with internal and external daughters were considered by subtracting the number of internal DE from the total and by using internal EBV instead of parent averages from EBV_MACE_ to compute external DRP. Based on Legarra et al. [13], Gengler and Vanderick [16] integrated MACE information into the official Walloon genetic evaluation for milk production. External EBV were estimated by selection index theory and internal contributions were considered as in VanRaden and Tooker [17]. Thus, while these two latter approaches and the approach proposed in this study consider internal contributions to MACE information in a similar manner [See Additional file 2], the main advantage of the proposed approach is to avoid a pre-processing deregression step or computation of external EBV.

#### Internal animals

**Figure 2:**
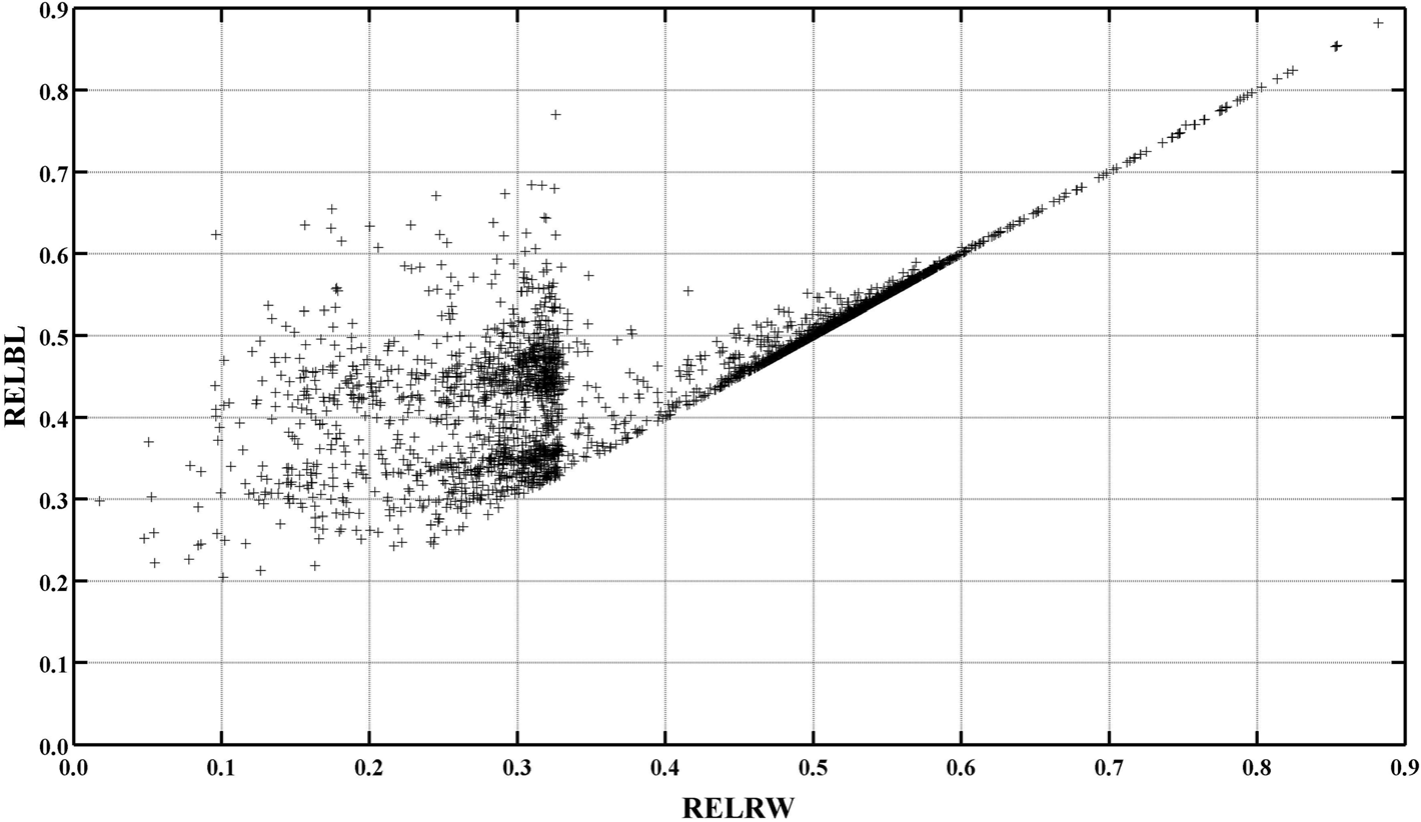
**Reliabilities for internal progeny.** Reliabilities associated with the Bayesian evaluation that considers double-counting of contributions due to relationships and due to records (REL_BL_) as a function of reliabilities associated with the official Walloon evaluation (REL_RW_) for the 3331 internal animals sired by internally used sires (i.e., having daughters with records in the Walloon Region) for milk yield.

The effect of the integration of MACE information on predictions was also studied for internal animals that were not associated with MACE information and that were sired by internally used sires. A total of 3331 internal animals was considered. If double-counting of contributions due to relationships and due to records were avoided (i.e. EVAL_BL_), integration of MACE information led to an increase of the REL from 0.32 to 0.42 for milk yield and from 0.31 to 0.42 for fat and protein yields for internal animals that had a REL_W_ less than 0.50 (Table 8). These increases were equivalent to 2.4 DE for milk yield, 2.3 DE for fat yield and 2.4 DE for protein yield. On average, no increase in REL was observed for internal animals with REL_W_ greater than 0.50 (Tables 9 and 10; Figure 2). Therefore, integration of MACE information was mostly relevant for external animals that were associated with this information and for internal animals with a low REL_W_ sired by external animals.

**Table 8:** **Parameters for internal animals with a Walloon reliability less than 0.50 and sired by internally used sires**

**Traits**	**N** ^**1**^	**Parameters** ^**2**^	**Genetic evaluation**				
			**EVAL**_**W**_	**EVAL**_**BLNN**_	**EVAL**_**BLRE**_	**EVAL**_**BLR**_	**EVAL**_**BL**_
Milk yield	1948	r	0.944	0.995	0.995	0.999	1.000
		RE_tot_	245.1	1655.2	1655.2	245.1	245.1
		REL	0.32 (0.10)	0.57 (0.06)	0.56 (0.06)	0.43 (0.07)	0.42 (0.07)
Fat yield	1694	r	0.923	0.994	0.994	0.999	1.000
		RE_tot_	102.6	1254.9	1254.9	102.6	102.6
		REL	0.31 (0.09)	0.56 (0.06)	0.56 (0.06)	0.42 (0.08)	0.42 (0.08)
Protein yield	1786	r	0.938	0.995	0.995	0.999	1.000
		RE_tot_	148.4	1243.5	1243.5	148.4	148.4
		REL	0.31 (0.09)	0.56 (0.06)	0.56 (0.06)	0.42 (0.08)	0.42 (0.08)

^1^N = Number of internal animals; ^2^r = rank correlation between EVAL_BL_ and EVAL_W_, EVAL_BLNN_, EVAL_BLRE_ or EVAL_BLR_; RE_tot_ = Total amount of record equivalents; REL = average reliability (SD in parentheses).

**Table 9:** **Parameters for internal animals with a Walloon reliability between 0.50 and 0.74 and sired by internally used sires**

**Traits**	**N** ^**1**^	**Parameters** ^**2**^	**Genetic evaluation**				
			**EVAL**_**W**_	**EVAL**_**BLNN**_	**EVAL**_**BLRE**_	**EVAL**_**BLR**_	**EVAL**_**BL**_
Milk yield	1360	r	0.999	>0.999	>0.999	>0.999	1.000
		RE_tot_	1205.7	2759.1	2759.1	1205.7	1205.7
		REL	0.55 (0.04)	0.67 (0.02)	0.67 (0.03)	0.55 (0.03)	0.55 (0.03)
Fat yield	1607	r	0.999	>0.999	>0.999	>0.999	1.000
		RE_tot_	1322.0	3125.6	3125.6	1322.0	1322.0
		REL	0.57 (0.04)	0.68 (0.03)	0.68 (0.03)	0.57 (0.04)	0.57 (0.04)
Protein yield	1516	r	0.999	>0.999	>0.999	>0.999	1.000
		RE_tot_	1252.0	2787.7	2787.7	1252.0	1252.0
		REL	0.56 (0.04)	0.68 (0.03)	0.68 (0.03)	0.56 (0.04)	0.56 (0.04)

^1^N = Number of internal animals; ^2^r = rank correlation between EVAL_BL_ and EVAL_W_, EVAL_BLNN_, EVAL_BLRE_ or EVAL_BLR_; RE_tot_ = Total amount of record equivalents; REL = average reliability (SD in parentheses).

**Table 10:** **Parameters for internal animals with a Walloon reliability greater than 0.74 and sired by internally used sires**

**Traits**	**N** ^**1**^	**Parameters** ^**2**^	**Genetic evaluation**				
			**EVAL**_**W**_	**EVAL**_**BLNN**_	**EVAL**_**BLRE**_	**EVAL**_**BLR**_	**EVAL**_**BL**_
Milk yield	23	r	0.998	0.999	1.000	0.999	1.000
		RE_tot_	132.6	156.9	156.9	132.6	132.6
		REL	0.80 (0.04)	0.82 (0.03)	0.82 (0.03)	0.80 (0.04)	0.80 (0.04)
Fat yield	30	r	0.999	1.000	>0.999	1.000	1.000
		RE_tot_	158.8	190.6	190.6	158.8	158.8
		REL	0.81 (0.04)	0.83 (0.03)	0.83 (0.03)	0.81 (0.04)	0.81 (0.04)
Protein yield	29	r	0.999	>0.999	>0.999	1.000	1.000
		RE_tot_	147.7	174.6	174.6	147.7	147.7
		REL	0.80 (0.04)	0.83 (0.03)	0.83 (0.03)	0.80 (0.04)	0.80 (0.04)

^1^N = Number of internal animals; ^2^r = rank correlation between EVAL_BL_ and EVAL_W_, EVAL_BLNN_, EVAL_BLRE_ or EVAL_BLR_; RE_tot_ = Total amount of record equivalents; REL = average reliability (SD in parentheses).

The effect of double-counting was also studied in comparison to EVAL_BL_ for the 3331 internal animals that were only associated with Walloon information and that were sired by internally used sires. Own contributions due to relationships for internal animals with REL_W_ less than 0.50 represented from 85.2% of the total contributions for milk yield to 91.8% for fat yield (Table 8). These percentages ranged from 55.1% for protein yield to 57.7% for fat yield for internal animals with REL_W_ between 0.50 and 0.75, and from 15.4% for protein yield to 16.7% for fat yield for internal animals with REL_W_ greater than 0.75 (Tables 9 and 10). As stated before, these observations were as expected based on selection index theory [25], and double-counting of own contributions due to relationships was mostly present for internal animals with low REL_W_. However, internal animals were also affected by double-counting of contributions due to relationships and due to records that originated from their sires (and relatives) through the contributions due to relationships. Double-counting that originated from their own contributions and from their sires (and relatives) could be observed based on a comparison of REL_BLRE_, REL_BLR_ and REL_BL_ and of r between EBV_BL_ and EBV_BLRE_ or EBV_BLR_ (Tables 8, 9 and 10). Double-counting of contributions due to records that originated from sires of internal animals had minor effects on the average REL_BLR_ associated with internal animals (at most 1%) and rankings of internal animals (r ≥ 0.999; Tables 8, 9 and 10). However, double-counting of contributions due to relationships led to an increase of average REL by at least 0.14 points for internal animals with REL_W_ less than 0.50 and by at least 0.11 points for internal animals with REL_W_ ranging from 0.50 to 0.74. The increase of average REL was lower for internal animals with REL_W_ greater than 0.75 (>0.02 points; Tables 8, 9 and 10). Although the average REL_BLR_ and REL_BLRE_ were (slightly) overestimated for both evaluations, double-counting of contributions due to records and due to relationships had little effect on the ranking of internal animals compared to the ranking of EVAL_BL_, regardless of the group of internal animals or trait considered. Indeed, rank correlations between EVAL_BL_ and EVAL_BLR_ or EVAL_BLRE_ were greater than 0.99 (Tables 8, 9 and 10). All these results show that double-counting of contributions due to relationships and due to records can be ignored for the prediction of EBV for internal animals that are sired by external animals. However, all double-counting must be taken into account to estimate REL accurately.

#### On the implementation

Considering all groups of animals, i.e. internally used and unused sires, as well as internal animals sired by internally used sires, our results for the Walloon example suggest that contributions due to relationships can be ignored. Indeed, the different rank correlations for EVAL_BLRE_ (i.e. the Bayesian evaluation that took only double-counting of contributions due to records into account) were similar to the rank correlations of EVAL_BL_. Furthermore, in practice, the TSA could be difficult to apply if a high number of animals is associated with external information because it requires the inversion of a, potentially, dense matrix for each iteration. However, effects of double-counting of contributions due to relationships should be tested before ignoring it. For example, overestimation of REL could occur especially for traits for which contributions due to relationships would be at least as significant as contributions due to records (e.g., if the phenotypes are expensive to obtain). Furthermore, REL associated with the modified MME were estimated based on the inverted LHS. Although this was feasible for the simulated and Walloon data, this may not be feasible in most cases, and approaches that estimate REL (e.g., [15,18]) could be modified to take into account RE (or DE) associated with external information.

The Walloon example was considered as an evaluation that blends MACE and Walloon (internal) information in the context of official Walloon genetic evaluations for Holstein cattle. However, the Walloon example can also be considered as a particular case of an internal evaluation that has no internal data and blends only sources of external information, i.e. MACE and Walloon information, that are partially based on the same information, i.e. the Walloon information. This case can be extended to more general cases for which internal data may exist and external animals are associated with at least two sources of information (e.g., E_1_ and E_2_) that are partially based on the same external records or information. Double-counting of external information that is shared by the sources of external information, e.g. E_1_ and E_2_, can be avoided by the proposed approach thanks to the knowledge and availability of EBV and associated REL that are based only on external information that is shared by the sources of external information. Nevertheless, although taking external information that is shared by different sources of external information into consideration seems to be possible with the proposed approach, this may be difficult in practice because it requires that EBV and associated REL based on shared external information are known and available.

## Conclusions

The proposed unified method integrated and blended several sources of information into an internal genetic evaluation in an appropriate manner. The results also showed that the proposed method was able to avoid double-counting of contributions due to records and due to relationships. Furthermore, because all available external sources of information were correctly propagated, relatives of external animals benefited from integrated information and, therefore, received more reliable EBV. The unified method could also be used in the context of single-step genomic evaluations to integrate external information to indirectly recover a large amount of external phenotypic information [26]. While the simulated and Walloon examples were univariate, the unified method was developed for multi-trait models that, e.g., allow evaluation of only internally available traits (e.g., methane emissions, fine milk composition traits, such as fatty acids, milk proteins and other minor components), using additional external information from correlated traits (e.g., traits evaluated by Interbull).

## Additional files

Additional file 1
**Integration of two sources of external information into a genetic evaluation.** This file describes a derivation that integrates two sources of external information into a genetic evaluation, based on a Bayesian view of the mixed models [27] and similar to the Bayesian derivation of Legarra et al. [13] that integrates one source of external information into a genetic evaluation. Additional file 2
**Double-counting between internal and external information.** This file describes the development to avoid double-counting of contributions due to records between internal and external information.
