# Supplementary material for: Unified method to integrate and blend several, potentially related, sources of information for genetic evaluation
Source: Genet Sel Evol. 2014 Sep 30;46(1):59. doi: 10.1186/s12711-014-0059-3 (PMC4179859; doi:10.1186/s12711-014-0059-3)
Supplement: Additional file 1 — Integration of two sources of external information into a genetic evaluation. This file describes a derivation that integrates two sources of external information into a genetic evaluation, based on a Bayesian view of the mixed models [27] and similar to the Bayesian derivation of Legarra et al. [13] that integrates one source of external information into a genetic evaluation. [file 12711_2014_59_MOESM1_ESM.pdf]

Additional file 1

Integration of two sources of external information into a genetic evaluation

The following Bayesian derivation is similar to the Bayesian derivation of Legarra et al. [13] that integrates one source of external information into an internal genetic evaluation in the context of multi-breed genetic evaluations for beef cattle.

Assume a set of animals partitioned in four groups. The first group (i.e. internal animals $\mathrm{A}_{1,2}^{0}$) has only records in the internal data set ($\mathbf{y}_{\mathrm{E}_0}$). The second group (i.e. external animals A_1) has records in the external data set, $\mathbf{y}_{\mathrm{E}_1}$, and may have records in $\mathbf{y}_{\mathrm{E}_0}$. The third group (i.e. external animals A_2) has records in the external data set, $\mathbf{y}_{\mathrm{E}_2}$, and may have records in $\mathbf{y}_{\mathrm{E}_0}$. The fourth group (i.e. external animals $\mathrm{A}_{1,2}$) have records in both $\mathbf{y}_{\mathrm{E}_1}$ and $\mathbf{y}_{\mathrm{E}_2}$, and may have also records in $\mathbf{y}_{\mathrm{E}_0}$. For the following genetic evaluations, variance components are assumed to be identical.

Concerning the notation of matrices in the following development (e.g., $\mathbf{X}_{\mathrm{E}_i(\mathrm{A}_l)}$), the subscript E_i refers to the ith source of data and the subscript within brackets (A_l) refers to the lth group of animals, respectively.

Assume a hypothetical joint genetic evaluation (denoted by the subscript J) of all animals ($\mathrm{A}_{1,2}^{0}$, A_1 , A_2 , $\mathrm{A}_{1,2}$) including both datasets $\mathbf{y}_{\mathrm{E}_1}$ and $\mathbf{y}_{\mathrm{E}_2}$. Because it was assumed that $\mathbf{y}_{\mathrm{E}_1}$ and $\mathbf{y}_{\mathrm{E}_2}$ were pre-corrected for fixed effects, the model partitioned among the four groups of animals can be written as:

$$\begin{bmatrix} \mathbf{y}_{\mathrm{E}_1(\mathrm{A}_1)} \\ \mathbf{y}_{\mathrm{E}_1(\mathrm{A}_{1,2})} \\ \mathbf{y}_{\mathrm{E}_2(\mathrm{A}_2)} \\ \mathbf{y}_{\mathrm{E}_2(\mathrm{A}_{1,2})} \end{bmatrix} = \begin{bmatrix} \mathbf{0} & \mathbf{Z}_{\mathrm{E}_1(\mathrm{A}_1)} & \mathbf{0} & \mathbf{0} \\ \mathbf{0} & \mathbf{0} & \mathbf{0} & \mathbf{Z}_{\mathrm{E}_1(\mathrm{A}_{1,2})} \\ \mathbf{0} & \mathbf{0} & \mathbf{Z}_{\mathrm{E}_2(\mathrm{A}_2)} & \mathbf{0} \\ \mathbf{0} & \mathbf{0} & \mathbf{0} & \mathbf{Z}_{\mathrm{E}_2(\mathrm{A}_{1,2})} \end{bmatrix} \begin{bmatrix} \mathbf{u}_{\mathrm{J}(\mathrm{A}_{1,2}^{0})} \\ \mathbf{u}_{\mathrm{J}(\mathrm{A}_1)} \\ \mathbf{u}_{\mathrm{J}(\mathrm{A}_2)} \\ \mathbf{u}_{\mathrm{J}(\mathrm{A}_{1,2})} \end{bmatrix} + \begin{bmatrix} \mathbf{e}_{\mathrm{J}_1(\mathrm{A}_1)} \\ \mathbf{e}_{\mathrm{J}_1(\mathrm{A}_{1,2})} \\ \mathbf{e}_{\mathrm{J}_2(\mathrm{A}_2)} \\ \mathbf{e}_{\mathrm{J}_2(\mathrm{A}_{1,2})} \end{bmatrix}$$

where $\mathbf{u}_J = \begin{bmatrix} \mathbf{u}'_{J(A^0_{1,2})} & \mathbf{u}'_{J(A_1)} & \mathbf{u}'_{J(A_2)} & \mathbf{u}'_{J(A_{1,2})} \end{bmatrix}'$ is the vector of genetic random effects for animals $A^0_{1,2}$, A_1, A_2 and $A_{1,2}$ for the evaluation J, $\mathbf{Z}_{E_1(A_1)}$ and $\mathbf{Z}_{E_1(A_{1,2})}$ are incidence matrices relating records of $\mathbf{y}_{E_1(A_1)}$ and $\mathbf{y}_{E_1(A_{1,2})}$ to $\mathbf{u}_{J(A_1)}$ and $\mathbf{u}_{J(A_{1,2})}$, respectively, and $\mathbf{Z}_{E_2(A_2)}$ and $\mathbf{Z}_{E_2(A_{1,2})}$ are incidence matrices relating records of $\mathbf{y}_{E_2(A_2)}$ and $\mathbf{y}_{E_2(A_{1,2})}$ to $\mathbf{u}_{J(A_2)}$ and $\mathbf{u}_{J(A_{1,2})}$, respectively, and $\mathbf{e}_{J_1}$ and $\mathbf{e}_{J_2}$ are the vectors of residuals associated with $\mathbf{y}_{E_1}$ and $\mathbf{y}_{E_2}$, respectively.

The corresponding mixed model equations (MME) can be written as:

$$
\begin{bmatrix}
\mathbf{G}_{E_0}^{(A^0_{1,2}A^0_{1,2})} & \mathbf{G}_{E_0}^{(A^0_{1,2}A_1)} & \mathbf{G}_{E_0}^{(A^0_{1,2}A_2)} & \mathbf{G}_{E_0}^{(A^0_{1,2}A_{1,2})} \\
\mathbf{G}_{E_0}^{(A_1A^0_{1,2})} & \mathbf{Z'}_{E_1(A_1)}\mathbf{R}_{E_1}^{(A_1A_1)}\mathbf{Z}_{E_1(A_1)} + \mathbf{G}_{E_0}^{(A_1A_1)} & \mathbf{G}_{E_0}^{(A_1A_2)} & \mathbf{Z'}_{E_1(A_1)}\mathbf{R}_{E_1}^{(A_1A_{1,2})}\mathbf{Z}_{E_1(A_{1,2})} + \mathbf{G}_{E_0}^{(A_1A_{1,2})} \\
\mathbf{G}_{E_0}^{(A_2A^0_{1,2})} & \mathbf{G}_{E_0}^{(A_2A_1)} & \mathbf{Z'}_{E_2(A_2)}\mathbf{R}_{E_2}^{(A_2A_2)}\mathbf{Z}_{E_2(A_2)} + \mathbf{G}_{E_0}^{(A_2A_2)} & \mathbf{Z'}_{E_2(A_2)}\mathbf{R}_{E_2}^{(A_2A_{1,2})}\mathbf{Z}_{E_2(A_{1,2})} + \mathbf{G}_{E_0}^{(A_2A_{1,2})} \\
\mathbf{G}_{E_0}^{(A_{1,2}A^0_{1,2})} & \mathbf{Z'}_{E_1(A_{1,2})}\mathbf{R}_{E_1}^{(A_{1,2}A_1)}\mathbf{Z}_{E_1(A_1)} + \mathbf{G}_{E_0}^{(A_{1,2}A_1)} & \mathbf{Z'}_{E_2(A_{1,2})}\mathbf{R}_{E_2}^{(A_{1,2}A_2)}\mathbf{Z}_{E_2(A_2)} + \mathbf{G}_{E_0}^{(A_{1,2}A_2)} & \mathbf{Z'}_{E_1(A_{1,2})}\mathbf{R}_{E_1}^{(A_{1,2}A_{1,2})}\mathbf{Z}_{E_1(A_{1,2})} + \mathbf{Z'}_{E_2(A_{1,2})}\mathbf{R}_{E_2}^{(A_{1,2}A_{1,2})}\mathbf{Z}_{E_2(A_{1,2})} + \mathbf{G}_{E_0}^{(A_{1,2}A_{1,2})}
\end{bmatrix}
\begin{bmatrix}
\hat{\mathbf{u}}_{J(A^0_{1,2})} \\
\hat{\mathbf{u}}_{J(A_1)} \\
\hat{\mathbf{u}}_{J(A_2)} \\
\hat{\mathbf{u}}_{J(A_{1,2})}
\end{bmatrix}
=
\begin{bmatrix}
\mathbf{0} \\
\mathbf{Z'}_{E_1(A_1)}\mathbf{R}_{E_1}^{(A_1A_1)}\mathbf{y}_{E_1(A_1)} \\
\mathbf{Z'}_{E_2(A_2)}\mathbf{R}_{E_2}^{(A_2A_2)}\mathbf{y}_{E_2(A_2)} \\
\mathbf{Z'}_{E_1(A_{1,2})}\mathbf{R}_{E_1}^{(A_{1,2}A_{1,2})}\mathbf{y}_{E_1(A_{1,2})} + \mathbf{Z'}_{E_2(A_{1,2})}\mathbf{R}_{E_2}^{(A_{1,2}A_{1,2})}\mathbf{y}_{E_2(A_{1,2})}
\end{bmatrix}
$$

(equation 1.1)

where $\mathbf{G}_{E_0}^{-1} = \begin{bmatrix}
\mathbf{G}_{E_0}^{(A^0_{1,2}A^0_{1,2})} & \mathbf{G}_{E_0}^{(A^0_{1,2}A_1)} & \mathbf{G}_{E_0}^{(A^0_{1,2}A_2)} & \mathbf{G}_{E_0}^{(A^0_{1,2}A_{1,2})} \\
\mathbf{G}_{E_0}^{(A_1A^0_{1,2})} & \mathbf{G}_{E_0}^{(A_1A_1)} & \mathbf{G}_{E_0}^{(A_1A_2)} & \mathbf{G}_{E_0}^{(A_1A_{1,2})} \\
\mathbf{G}_{E_0}^{(A_2A^0_{1,2})} & \mathbf{G}_{E_0}^{(A_2A_1)} & \mathbf{G}_{E_0}^{(A_2A_2)} & \mathbf{G}_{E_0}^{(A_2A_{1,2})} \\
\mathbf{G}_{E_0}^{(A_{1,2}A^0_{1,2})} & \mathbf{G}_{E_0}^{(A_{1,2}A_1)} & \mathbf{G}_{E_0}^{(A_{1,2}A_2)} & \mathbf{G}_{E_0}^{(A_{1,2}A_{1,2})}
\end{bmatrix}$ is the inverse of the additive

(co)variance matrix including all animals and $\begin{bmatrix} \mathbf{R}_{E_1}^{(A_1A_1)} & \mathbf{R}_{E_1}^{(A_1A_{1,2})} \\ \mathbf{R}_{E_1}^{(A_{1,2}A_1)} & \mathbf{R}_{E_1}^{(A_{1,2}A_{1,2})} \end{bmatrix}$ and $\begin{bmatrix} \mathbf{R}_{E_2}^{(A_2A_2)} & \mathbf{R}_{E_2}^{(A_2A_{1,2})} \\ \mathbf{R}_{E_2}^{(A_{1,2}A_2)} & \mathbf{R}_{E_2}^{(A_{1,2}A_{1,2})} \end{bmatrix}$ are the inverse of the residual (co)variance matrices associated with $\mathbf{e}_{J_1}$ and $\mathbf{e}_{J_2}$, respectively.

Assume an internal genetic evaluation (denoted by the subscript E_0) of all animals (i.e. animals $A_{1,2}^0$, A_1, A_2 and $A_{1,2}$) including only $\mathbf{y}_{E_0}$ and using the prior distribution $p(\hat{\mathbf{u}}_{E_0} | \mathbf{y}_{E_1}, \mathbf{y}_{E_2}) = MVN(\boldsymbol{\mu}, \mathbf{G}^*)$ [27] where $\mathbf{G}^*$ is the inverse of the left-hand-side (LHS) of the equation 1.1 and $\boldsymbol{\mu}$ is the solutions of the equation 1.1. The model for the genetic evaluation E_0 can be written as:

$$\mathbf{y}_{E_0} = \mathbf{X}_{E_0}\boldsymbol{\beta}_{E_0} + \mathbf{Z}_{E_0}\mathbf{u}_{E_0} + \mathbf{e}_{E_0},$$

where $\mathbf{X}_{E_0}$ and $\mathbf{Z}_{E_0}$ are incidence matrices relating records in $\mathbf{y}_{E_0}$ to the vector of fixed effects $\boldsymbol{\beta}_{E_0}$ and the vector of genetic random effects $\mathbf{u}_{E_0} = \begin{bmatrix} \mathbf{u}'_{E_0(A_{1,2}^0)} & \mathbf{u}'_{E_0(A_1)} & \mathbf{u}'_{E_0(A_2)} & \mathbf{u}'_{E_0(A_{1,2})} \end{bmatrix}'$, respectively and $\mathbf{e}_{E_0}$ is the vector of residuals.

The MME can be written as:

$$\begin{bmatrix} \mathbf{X}'_{E_0}\mathbf{R}_{E_0}^{-1}\mathbf{X}_{E_0} & \mathbf{X}'_{E_0}\mathbf{R}_{E_0}^{-1}\mathbf{Z}_{E_0} \\ \mathbf{Z}'_{E_0}\mathbf{R}_{E_0}^{-1}\mathbf{X}_{E_0} & \mathbf{Z}'_{E_0}\mathbf{R}_{E_0}^{-1}\mathbf{Z}_{E_0} + \mathbf{G}^{*-1} \end{bmatrix} \begin{bmatrix} \hat{\boldsymbol{\beta}}_{E_0} \\ \hat{\mathbf{u}}_{E_0} \end{bmatrix} = \begin{bmatrix} \mathbf{X}'_{E_0}\mathbf{R}_{E_0}^{-1}\mathbf{y}_{E_0} \\ \mathbf{Z}'_{E_0}\mathbf{R}_{E_0}^{-1}\mathbf{y}_{E_0} + \mathbf{G}^{*-1}\boldsymbol{\mu} \end{bmatrix}, \quad \text{(equation 1.2)}$$

where $\mathbf{R}_{E_0}^{-1}$ is the inverse of the residual (co)variance matrix associated with $\mathbf{e}_{E_0}$.

However, the evaluation J (equation 1.1), and therefore $\mathbf{G}^*$ and $\boldsymbol{\mu}$, are unknown.

Assume that two genetic evaluations (denoted by the subscripts E_1 and E_2, respectively) for two groups of external animals (i.e. animals A_1 and $A_{1,2}$, and animals A_2 and $A_{1,2}$, respectively)

which do not include in the genealogy internal animals (i.e. animals $A_{1,2}^{0}$ and A_2, and animals $A_{1,2}^{0}$ and A_1, respectively) are known. The model for the genetic evaluation E_1 of only external animals A_1 and $A_{1,2}$ including only $\mathbf{y}_{E_1}$ and which does not include in the genealogy animals $A_{1,2}^{0}$ and A_2 can be written as:

$$\begin{bmatrix} \mathbf{y}_{E_1(A_1)} \\ \mathbf{y}_{E_1(A_{1,2})} \end{bmatrix} = \begin{bmatrix} \mathbf{Z}_{E_1(A_1)} & \mathbf{0} \\ \mathbf{0} & \mathbf{Z}_{E_1(A_{1,2})} \end{bmatrix} \begin{bmatrix} \mathbf{u}_{E_1(A_1)} \\ \mathbf{u}_{E_1(A_{1,2})} \end{bmatrix} + \begin{bmatrix} \mathbf{e}_{E_1(A_1)} \\ \mathbf{e}_{E_1(A_{1,2})} \end{bmatrix},$$

where $\mathbf{u}_{E_1(A_1)}$ and $\mathbf{u}_{E_1(A_{1,2})}$ are the vectors of genetic random effects for animals A_1 and $A_{1,2}$ for the genetic evaluation E_1.

The MME can be written as:

$$\begin{bmatrix} \mathbf{Z}'_{E_1(A_1)} \mathbf{R}_{E_1}^{(A_1A_1)} \mathbf{Z}_{E_1(A_1)} + \mathbf{G}_{E_1}^{*(A_1A_1)} & \mathbf{Z}'_{E_1(A_1)} \mathbf{R}_{E_1}^{(A_1A_{1,2})} \mathbf{Z}_{E_1(A_{1,2})} + \mathbf{G}_{E_1}^{*(A_1A_{1,2})} \\ \mathbf{Z}'_{E_1(A_{1,2})} \mathbf{R}_{E_1}^{(A_{1,2}A_1)} \mathbf{Z}_{E_1(A_1)} + \mathbf{G}_{E_1}^{*(A_{1,2}A_1)} & \mathbf{Z}'_{E_1(A_{1,2})} \mathbf{R}_{E_1}^{(A_{1,2}A_{1,2})} \mathbf{Z}_{E_1(A_{1,2})} + \mathbf{G}_{E_1}^{*(A_{1,2}A_{1,2})} \end{bmatrix} \begin{bmatrix} \hat{\mathbf{u}}_{E_1(A_1)} \\ \hat{\mathbf{u}}_{E_1(A_{1,2})} \end{bmatrix}$$

(equation 1.3)

$$= \begin{bmatrix} \mathbf{Z}'_{E_1(A_1)} \mathbf{R}_{E_1}^{(A_1A_1)} \mathbf{y}_{E_1(A_1)} \\ \mathbf{Z}'_{E_1(A_{1,2})} \mathbf{R}_{E_1}^{(A_{1,2}A_{1,2})} \mathbf{y}_{E_1(A_{1,2})} \end{bmatrix} = \mathbf{D}_{E_1}^{*-1} \begin{bmatrix} \hat{\mathbf{u}}_{E_1(A_1)} \\ \hat{\mathbf{u}}_{E_1(A_{1,2})} \end{bmatrix}$$

where $\mathbf{G}_{E_1}^{*-1} = \begin{bmatrix} \mathbf{G}_{E_1}^{*(A_1A_1)} & \mathbf{G}_{E_1}^{*(A_1A_{1,2})} \\ \mathbf{G}_{E_1}^{*(A_{1,2}A_1)} & \mathbf{G}_{E_1}^{*(A_{1,2}A_{1,2})} \end{bmatrix} = \begin{bmatrix} \mathbf{G}_{E_1(A_1A_1)} & \mathbf{G}_{E_1(A_1A_{1,2})} \\ \mathbf{G}_{E_1(A_{1,2}A_1)} & \mathbf{G}_{E_1(A_{1,2}A_{1,2})} \end{bmatrix}^{-1}$ is the inverse of the additive genetic (co)variance matrix for the external genetic evaluation E_1.

Similarly, the MME for the genetic evaluation E_2 of only external animals A_2 and $A_{1,2}$ including only $\mathbf{y}_{E_2}$ and which does not include in genealogy animals $A_{1,2}^{0}$ and A_1 can be written as:

$$\begin{bmatrix} \mathbf{Z}'_{E_2(A_2)}\mathbf{R}_{E_2}^{(A_2A_2)}\mathbf{Z}_{E_2(A_2)} + \mathbf{G}_{E_2}^{*(A_2A_2)} & \mathbf{Z}'_{E_2(A_2)}\mathbf{R}_{E_2}^{(A_2A_{1,2})}\mathbf{Z}_{E_2(A_{1,2})} + \mathbf{G}_{E_2}^{*(A_2A_{1,2})} \\ \mathbf{Z}'_{E_2(A_{1,2})}\mathbf{R}_{E_2}^{(A_{1,2}A_2)}\mathbf{Z}_{E_2(A_2)} + \mathbf{G}_{E_2}^{*(A_{1,2}A_2)} & \mathbf{Z}'_{E_2(A_{1,2})}\mathbf{R}_{E_2}^{(A_{1,2}A_{1,2})}\mathbf{Z}_{E_2(A_{1,2})} + \mathbf{G}_{E_2}^{*(A_{1,2}A_{1,2})} \end{bmatrix} \begin{bmatrix} \hat{\mathbf{u}}_{E_2(A_2)} \\ \hat{\mathbf{u}}_{E_2(A_{1,2})} \end{bmatrix}$$

(equation 1.4)

$$= \begin{bmatrix} \mathbf{Z}'_{E_2(A_2)}\mathbf{R}_{E_2}^{(A_2A_2)}\mathbf{y}_{E_2(A_2)} \\ \mathbf{Z}'_{E_2(A_{1,2})}\mathbf{R}_{E_2}^{(A_{1,2}A_{1,2})}\mathbf{y}_{E_2(A_{1,2})} \end{bmatrix} = \mathbf{D}_{E_2}^{*-1} \begin{bmatrix} \hat{\mathbf{u}}_{E_2(A_2)} \\ \hat{\mathbf{u}}_{E_2(A_{1,2})} \end{bmatrix}$$

where $\hat{\mathbf{u}}_{E_2(A_2)}$ and $\hat{\mathbf{u}}_{E_2(A_{1,2})}$ are the vectors of genetic random effects for animals A_2 and $A_{1,2}$ for the genetic evaluation E_2, and $\mathbf{G}_{E_2}^{*-1} = \begin{bmatrix} \mathbf{G}_{E_2}^{*(A_2A_2)} & \mathbf{G}_{E_2}^{*(A_2A_{1,2})} \\ \mathbf{G}_{E_2}^{*(A_{1,2}A_2)} & \mathbf{G}_{E_2}^{*(A_{1,2}A_{1,2})} \end{bmatrix} = \begin{bmatrix} \mathbf{G}_{E_2(A_2A_2)} & \mathbf{G}_{E_2(A_2A_{1,2})} \\ \mathbf{G}_{E_2(A_{1,2}A_2)} & \mathbf{G}_{E_2(A_{1,2}A_{1,2})} \end{bmatrix}^{-1}$ is the additive genetic (co)variance matrix for the genetic evaluation E_2.

Therefore,

$$\mathbf{D}_{E_1}^{*-1} - \mathbf{G}_{E_1}^{*-1} = \begin{bmatrix} \mathbf{D}_{E_1}^{*(A_1A_1)} & \mathbf{D}_{E_1}^{*(A_1A_{1,2})} \\ \mathbf{D}_{E_1}^{*(A_{1,2}A_1)} & \mathbf{D}_{E_1}^{*(A_{1,2}A_{1,2})} \end{bmatrix} - \begin{bmatrix} \mathbf{G}_{E_1}^{*(A_1A_1)} & \mathbf{G}_{E_1}^{*(A_1A_{1,2})} \\ \mathbf{G}_{E_1}^{*(A_{1,2}A_1)} & \mathbf{G}_{E_1}^{*(A_{1,2}A_{1,2})} \end{bmatrix}$$

(equation 1.5)

$$= \begin{bmatrix} \mathbf{Z}'_{E_1(A_1)}\mathbf{R}_{E_1}^{(A_1A_1)}\mathbf{Z}_{E_1(A_1)} & \mathbf{Z}'_{E_1(A_1)}\mathbf{R}_{E_1}^{(A_1A_{1,2})}\mathbf{Z}_{E_1(A_{1,2})} \\ \mathbf{Z}'_{E_1(A_{1,2})}\mathbf{R}_{E_1}^{(A_{1,2}A_1)}\mathbf{Z}_{E_1(A_1)} & \mathbf{Z}'_{E_1(A_{1,2})}\mathbf{R}_{E_1}^{(A_{1,2}A_{1,2})}\mathbf{Z}_{E_1(A_{1,2})} \end{bmatrix}$$

and, similarly,

$$\mathbf{D}_{E_2}^{*-1} - \mathbf{G}_{E_2}^{*-1} = \begin{bmatrix} \mathbf{Z}'_{E_2(A_2)}\mathbf{R}_{E_2}^{(A_2A_2)}\mathbf{Z}_{E_2(A_2)} & \mathbf{Z}'_{E_2(A_2)}\mathbf{R}_{E_2}^{(A_2A_{1,2})}\mathbf{Z}_{E_2(A_{1,2})} \\ \mathbf{Z}'_{E_2(A_{1,2})}\mathbf{R}_{E_2}^{(A_{1,2}A_2)}\mathbf{Z}_{E_2(A_2)} & \mathbf{Z}'_{E_2(A_{1,2})}\mathbf{R}_{E_2}^{(A_{1,2}A_{1,2})}\mathbf{Z}_{E_2(A_{1,2})} \end{bmatrix} \quad \text{(equation 1.6).}$$

Substituting "unknown" terms of the equation 1.1 by their corresponding terms from the equations 1.3, 1.4, 1.5 and 1.6, the MME (equation 1.1) can be written as:

$$
\begin{bmatrix}
\mathbf{G}_{E_0}^{\left(A_{1,2}^0 A_{1,2}^0\right)} & \mathbf{G}_{E_0}^{\left(A_{1,2}^0 A_1\right)} & \mathbf{G}_{E_0}^{\left(A_{1,2}^0 A_2\right)} & \mathbf{G}_{E_0}^{\left(A_{1,2}^0 A_{1,2}\right)} \\
\mathbf{G}_{E_0}^{\left(A_1 A_{1,2}^0\right)} & \mathbf{D}_{E_1}^{*\left(A_1 A_1\right)} - \mathbf{G}_{E_1}^{*\left(A_1 A_1\right)} + \mathbf{G}_{E_0}^{\left(A_1 A_1\right)} & \mathbf{G}_{E_0}^{\left(A_1 A_2\right)} & \mathbf{D}_{E_1}^{*\left(A_1 A_{1,2}\right)} - \mathbf{G}_{E_1}^{*\left(A_1 A_{1,2}\right)} + \mathbf{G}_{E_0}^{\left(A_1 A_{1,2}\right)} \\
\mathbf{G}_{E_0}^{\left(A_2 A_{1,2}^0\right)} & \mathbf{G}_{E_0}^{\left(A_2 A_1\right)} & \mathbf{D}_{E_2}^{*\left(A_2 A_2\right)} - \mathbf{G}_{E_2}^{*\left(A_2 A_2\right)} + \mathbf{G}_{E_0}^{\left(A_2 A_2\right)} & \mathbf{D}_{E_2}^{*\left(A_2 A_{1,2}\right)} - \mathbf{G}_{E_2}^{*\left(A_2 A_{1,2}\right)} + \mathbf{G}_{E_0}^{\left(A_2 A_{1,2}\right)} \\
\mathbf{G}_{E_0}^{\left(A_{1,2} A_{1,2}^0\right)} & \mathbf{D}_{E_1}^{*\left(A_{1,2} A_1\right)} - \mathbf{G}_{E_1}^{*\left(A_{1,2} A_1\right)} + \mathbf{G}_{E_0}^{\left(A_{1,2} A_1\right)} & \mathbf{D}_{E_2}^{*\left(A_{1,2} A_2\right)} - \mathbf{G}_{E_2}^{*\left(A_{1,2} A_2\right)} + \mathbf{G}_{E_0}^{\left(A_{1,2} A_2\right)} & \begin{matrix} \mathbf{D}_{E_1}^{*\left(A_{1,2} A_{1,2}\right)} - \mathbf{G}_{E_1}^{*\left(A_{1,2} A_{1,2}\right)} + \\ \mathbf{D}_{E_2}^{*\left(A_{1,2} A_{1,2}\right)} - \mathbf{G}_{E_2}^{*\left(A_{1,2} A_{1,2}\right)} + \mathbf{G}_{E_0}^{\left(A_{1,2} A_{1,2}\right)} \end{matrix}
\end{bmatrix}
$$

$$
\begin{bmatrix}
\hat{\mathbf{u}}_{J\left(A_{1,2}^0\right)} \\
\hat{\mathbf{u}}_{J\left(A_1\right)} \\
\hat{\mathbf{u}}_{J\left(A_2\right)} \\
\hat{\mathbf{u}}_{J\left(A_{1,2}\right)}
\end{bmatrix}
=
\begin{bmatrix}
\mathbf{0} \\
\mathbf{D}_{E_1}^{*\left(A_1 A_1\right)} \hat{\mathbf{u}}_{E_1\left(A_1\right)} + \mathbf{D}_{E_1}^{*\left(A_1 A_{1,2}\right)} \hat{\mathbf{u}}_{E_1\left(A_{1,2}\right)} \\
\mathbf{D}_{E_2}^{*\left(A_2 A_2\right)} \hat{\mathbf{u}}_{E_2\left(A_2\right)} + \mathbf{D}_{E_2}^{*\left(A_2 A_{1,2}\right)} \hat{\mathbf{u}}_{E_2\left(A_{1,2}\right)} \\
\mathbf{D}_{E_1}^{*\left(A_{1,2} A_1\right)} \hat{\mathbf{u}}_{E_1\left(A_1\right)} + \mathbf{D}_{E_1}^{*\left(A_{1,2} A_{1,2}\right)} \hat{\mathbf{u}}_{E_1\left(A_{1,2}\right)} + \mathbf{D}_{E_2}^{*\left(A_{1,2} A_2\right)} \hat{\mathbf{u}}_{E_2\left(A_2\right)} + \mathbf{D}_{E_2}^{*\left(A_{1,2} A_{1,2}\right)} \hat{\mathbf{u}}_{E_2\left(A_{1,2}\right)}
\end{bmatrix}
$$

(equation 1.7).

By replacing $\mathbf{G}^{*-1}$ in the equation 1.2 by the LHS of the equation 1.7 and $\mathbf{G}^{*-1}\boldsymbol{\mu}$ by the RHS of the equation 1.7, the following equations are obtained:

$$
\begin{bmatrix}
\mathbf{X'}_{E_0}\mathbf{R}_{E_0}^{-1}\mathbf{X}_{E_0} & \mathbf{X'}_{E_0}\mathbf{R}_{E_0}^{-1}\mathbf{Z}_{E_0} \\
\mathbf{Z'}_{E_0}\mathbf{R}_{E_0}^{-1}\mathbf{X}_{E_0} & \mathbf{Z'}_{E_0}\mathbf{R}_{E_0}^{-1}\mathbf{Z}_{E_0} + \begin{bmatrix}
\mathbf{G}_{E_0}^{\left(A_{1,2}^0 A_{1,2}^0\right)} & \mathbf{G}_{E_0}^{\left(A_{1,2}^0 A_1\right)} & \mathbf{G}_{E_0}^{\left(A_{1,2}^0 A_2\right)} & \mathbf{G}_{E_0}^{\left(A_{1,2}^0 A_{1,2}\right)} \\
\mathbf{G}_{E_0}^{\left(A_1 A_{1,2}^0\right)} & \mathbf{D}_{E_1}^{*\left(A_1 A_1\right)} - \mathbf{G}_{E_1}^{*\left(A_1 A_1\right)} + \mathbf{G}_{E_0}^{\left(A_1 A_1\right)} & \mathbf{G}_{E_0}^{\left(A_1 A_2\right)} & \mathbf{D}_{E_1}^{*\left(A_1 A_{1,2}\right)} - \mathbf{G}_{E_1}^{*\left(A_1 A_{1,2}\right)} + \mathbf{G}_{E_0}^{\left(A_1 A_{1,2}\right)} \\
\mathbf{G}_{E_0}^{\left(A_2 A_{1,2}^0\right)} & \mathbf{G}_{E_0}^{\left(A_2 A_1\right)} & \mathbf{D}_{E_2}^{*\left(A_2 A_2\right)} - \mathbf{G}_{E_2}^{*\left(A_2 A_2\right)} + \mathbf{G}_{E_0}^{\left(A_2 A_2\right)} & \mathbf{D}_{E_2}^{*\left(A_2 A_{1,2}\right)} - \mathbf{G}_{E_2}^{*\left(A_2 A_{1,2}\right)} + \mathbf{G}_{E_0}^{\left(A_2 A_{1,2}\right)} \\
\mathbf{G}_{E_0}^{\left(A_{1,2} A_{1,2}^0\right)} & \mathbf{D}_{E_1}^{*\left(A_{1,2} A_1\right)} - \mathbf{G}_{E_1}^{*\left(A_{1,2} A_1\right)} + \mathbf{G}_{E_0}^{\left(A_{1,2} A_1\right)} & \mathbf{D}_{E_2}^{*\left(A_{1,2} A_2\right)} - \mathbf{G}_{E_2}^{*\left(A_{1,2} A_2\right)} + \mathbf{G}_{E_0}^{\left(A_{1,2} A_2\right)} & \begin{matrix} \mathbf{D}_{E_1}^{*\left(A_{1,2} A_{1,2}\right)} - \mathbf{G}_{E_1}^{*\left(A_{1,2} A_{1,2}\right)} + \\ \mathbf{D}_{E_2}^{*\left(A_{1,2} A_{1,2}\right)} - \mathbf{G}_{E_2}^{*\left(A_{1,2} A_{1,2}\right)} + \\ \mathbf{G}_{E_0}^{\left(A_{1,2} A_{1,2}\right)} \end{matrix}
\end{bmatrix}
\end{bmatrix}
\begin{bmatrix}
\hat{\boldsymbol{\beta}}_{E_0} \\
\hat{\mathbf{u}}_{E_0}
\end{bmatrix}
$$

$$
=
\begin{bmatrix}
\mathbf{X'}_{E_0}\mathbf{R}_{E_0}^{-1}\mathbf{y}_{E_0} \\
\mathbf{Z'}_{E_0}\mathbf{R}_{E_0}^{-1}\mathbf{y}_{E_0} + \begin{bmatrix}
\mathbf{0} \\
\mathbf{D}_{E_1}^{*\left(A_1 A_1\right)} \hat{\mathbf{u}}_{E_1\left(A_1\right)} + \mathbf{D}_{E_1}^{*\left(A_1 A_{1,2}\right)} \hat{\mathbf{u}}_{E_1\left(A_{1,2}\right)} \\
\mathbf{D}_{E_2}^{*\left(A_2 A_2\right)} \hat{\mathbf{u}}_{E_2\left(A_2\right)} + \mathbf{D}_{E_2}^{*\left(A_2 A_{1,2}\right)} \hat{\mathbf{u}}_{E_2\left(A_{1,2}\right)} \\
\mathbf{D}_{E_1}^{*\left(A_{1,2} A_1\right)} \hat{\mathbf{u}}_{E_1\left(A_1\right)} + \mathbf{D}_{E_1}^{*\left(A_{1,2} A_{1,2}\right)} \hat{\mathbf{u}}_{E_1\left(A_{1,2}\right)} + \mathbf{D}_{E_2}^{*\left(A_{1,2} A_2\right)} \hat{\mathbf{u}}_{E_2\left(A_2\right)} + \mathbf{D}_{E_2}^{*\left(A_{1,2} A_{1,2}\right)} \hat{\mathbf{u}}_{E_2\left(A_{1,2}\right)}
\end{bmatrix}
\end{bmatrix}
$$

(equation 1.8).

To simplify the system of equations 1.8, two genetic evaluations including in genealogy all animals (i.e. animals $A_{1,2}^0$, A_1 , A_2 and $A_{1,2}$) equivalent to the genetic evaluations E_1 and E_2 for the external animals can be performed. Therefore, the following model of a genetic evaluation of all animals including only $\mathbf{y}_{E_1}$ can be written as:

$$\begin{bmatrix} \mathbf{y}_{E_1(A_1)} \\ \mathbf{y}_{E_1(A_{1,2})} \end{bmatrix} = \begin{bmatrix} \mathbf{0} & \mathbf{Z}_{E_1(A_1)} & \mathbf{0} & \mathbf{0} \\ \mathbf{0} & \mathbf{0} & \mathbf{0} & \mathbf{Z}_{E_1(A_{1,2})} \end{bmatrix} \begin{bmatrix} \mathbf{u}_{E_1(A_{1,2}^0)} \\ \mathbf{u}_{E_1(A_1)} \\ \mathbf{u}_{E_1(A_2)} \\ \mathbf{u}_{E_1(A_{1,2})} \end{bmatrix} + \begin{bmatrix} \mathbf{e}_{E_1(A_1)} \\ \mathbf{e}_{E_1(A_{1,2})} \end{bmatrix},$$

where $\mathbf{u}_{E_1(A_{1,2}^0)}$ and $\mathbf{u}_{E_1(A_2)}$ are the vectors of estimated random additive genetic effects for animals $A_{1,2}^0$ and A_2.

The corresponding MME can be written as follows:

$$\begin{bmatrix} \mathbf{G}_{E_1}^{(A_{1,2}^0A_{1,2}^0)} & \mathbf{G}_{E_1}^{(A_{1,2}^0A_1)} & \mathbf{G}_{E_1}^{(A_{1,2}^0A_2)} & \mathbf{G}_{E_1}^{(A_{1,2}^0A_{1,2})} \\ \mathbf{G}_{E_1}^{(A_1A_{1,2}^0)} & \mathbf{Z}'_{E_1(A_1)}\mathbf{R}_{E_1}^{(A_1A_1)}\mathbf{Z}_{E_1(A_1)} + \mathbf{G}_{E_1}^{(A_1A_1)} & \mathbf{G}_{E_1}^{(A_1A_2)} & \mathbf{Z}'_{E_1(A_1)}\mathbf{R}_{E_1}^{(A_1A_{1,2})}\mathbf{Z}_{E_1(A_{1,2})} + \mathbf{G}_{E_1}^{(A_1A_{1,2})} \\ \mathbf{G}_{E_1}^{(A_2A_{1,2}^0)} & \mathbf{G}_{E_1}^{(A_2A_1)} & \mathbf{G}_{E_1}^{(A_2A_2)} & \mathbf{G}_{E_1}^{(A_2A_{1,2})} \\ \mathbf{G}_{E_1}^{(A_{1,2}A_{1,2}^0)} & \mathbf{Z}'_{E_1(A_{1,2})}\mathbf{R}_{E_1}^{(A_{1,2}A_1)}\mathbf{Z}_{E_1(A_1)} + \mathbf{G}_{E_1}^{(A_{1,2}A_1)} & \mathbf{G}_{E_1}^{(A_{1,2}A_2)} & \mathbf{Z}'_{E_1(A_{1,2})}\mathbf{R}_{E_1}^{(A_{1,2}A_{1,2})}\mathbf{Z}_{E_1(A_{1,2})} + \mathbf{G}_{E_1}^{(A_{1,2}A_{1,2})} \end{bmatrix} \begin{bmatrix} \hat{\mathbf{u}}_{E_1(A_{1,2}^0)} \\ \hat{\mathbf{u}}_{E_1(A_1)} \\ \hat{\mathbf{u}}_{E_1(A_2)} \\ \hat{\mathbf{u}}_{E_1(A_{1,2})} \end{bmatrix} = \mathbf{D}_{E_1}^{-1}\hat{\mathbf{u}}_{E_1}$$

$$= \begin{bmatrix} \mathbf{0} \\ \mathbf{Z}'_{E_1(A_1)}\mathbf{R}_{E_1}^{(A_1A_1)}\mathbf{y}_{E_1(A_1)} \\ \mathbf{0} \\ \mathbf{Z}'_{E_1(A_{1,2})}\mathbf{R}_{E_1}^{(A_{1,2}A_{1,2})}\mathbf{y}_{E_1(A_{1,2})} \end{bmatrix}$$

(equation 1.9)

where $\mathbf{G}_{E_1}^{-1} = \begin{bmatrix} \mathbf{G}_{E_1}^{(A_{1,2}^0A_{1,2}^0)} & \mathbf{G}_{E_1}^{(A_{1,2}^0A_1)} & \mathbf{G}_{E_1}^{(A_{1,2}^0A_2)} & \mathbf{G}_{E_1}^{(A_{1,2}^0A_{1,2})} \\ \mathbf{G}_{E_1}^{(A_1A_{1,2}^0)} & \mathbf{G}_{E_1}^{(A_1A_1)} & \mathbf{G}_{E_1}^{(A_1A_2)} & \mathbf{G}_{E_1}^{(A_1A_{1,2})} \\ \mathbf{G}_{E_1}^{(A_2A_{1,2}^0)} & \mathbf{G}_{E_1}^{(A_2A_1)} & \mathbf{G}_{E_1}^{(A_2A_2)} & \mathbf{G}_{E_1}^{(A_2A_{1,2})} \\ \mathbf{G}_{E_1}^{(A_{1,2}A_{1,2}^0)} & \mathbf{G}_{E_1}^{(A_{1,2}A_1)} & \mathbf{G}_{E_1}^{(A_{1,2}A_2)} & \mathbf{G}_{E_1}^{(A_{1,2}A_{1,2})} \end{bmatrix}$ is the additive genetic (co)variance matrix taking into account all animals.

Similarly, MME for a genetic evaluation of all animals including only $\mathbf{y}_{E_2}$ can be written as:

$$\begin{bmatrix} \mathbf{G}_{E_2}^{\left(A^0_{1,2}A^0_{1,2}\right)} & \mathbf{G}_{E_2}^{\left(A^0_{1,2}A_1\right)} & \mathbf{G}_{E_2}^{\left(A^0_{1,2}A_2\right)} & \mathbf{G}_{E_2}^{\left(A^0_{1,2}A_{1,2}\right)} \\ \mathbf{G}_{E_2}^{\left(A_1A^0_{1,2}\right)} & \mathbf{G}_{E_2}^{\left(A_1A_1\right)} & \mathbf{G}_{E_2}^{\left(A_1A_2\right)} & \mathbf{G}_{E_2}^{\left(A_1A_{1,2}\right)} \\ \mathbf{G}_{E_2}^{\left(A_2A^0_{1,2}\right)} & \mathbf{G}_{E_2}^{\left(A_2A_1\right)} & \mathbf{Z'}_{E_2\left(A_2\right)}\mathbf{R}_{E_2}^{\left(A_2A_2\right)}\mathbf{Z}_{E_2\left(A_2\right)} + \mathbf{G}_{E_2}^{\left(A_2A_2\right)} & \mathbf{Z'}_{E_2\left(A_2\right)}\mathbf{R}_{E_2}^{\left(A_2A_{1,2}\right)}\mathbf{Z}_{E_2\left(A_{1,2}\right)} + \mathbf{G}_{E_2}^{\left(A_2A_{1,2}\right)} \\ \mathbf{G}_{E_2}^{\left(A_{1,2}A^0_{1,2}\right)} & \mathbf{G}_{E_2}^{\left(A_{1,2}A_1\right)} & \mathbf{Z'}_{E_2\left(A_{1,2}\right)}\mathbf{R}_{E_2}^{\left(A_{1,2}A_2\right)}\mathbf{Z}_{E_2\left(A_2\right)} + \mathbf{G}_{E_2}^{\left(A_{1,2}A_2\right)} & \mathbf{Z'}_{E_2\left(A_{1,2}\right)}\mathbf{R}_{E_2}^{\left(A_{1,2}A_{1,2}\right)}\mathbf{Z}_{E_2\left(A_{1,2}\right)} + \mathbf{G}_{E_2}^{\left(A_{1,2}A_{1,2}\right)} \end{bmatrix} \begin{bmatrix} \hat{\mathbf{u}}_{E_2\left(A^0_{1,2}\right)} \\ \hat{\mathbf{u}}_{E_2\left(A_1\right)} \\ \hat{\mathbf{u}}_{E_2\left(A_2\right)} \\ \hat{\mathbf{u}}_{E_2\left(A_{1,2}\right)} \end{bmatrix} = \mathbf{D}_{E_2}^{-1}\hat{\mathbf{u}}_{E_2}$$

$$= \begin{bmatrix} \mathbf{0} \\ \mathbf{Z'}_{E_2\left(A_1\right)}\mathbf{R}_{E_2}^{\left(A_1A_1\right)}\mathbf{y}_{E_2\left(A_1\right)} \\ \mathbf{0} \\ \mathbf{Z'}_{E_2\left(A_{1,2}\right)}\mathbf{R}_{E_2}^{\left(A_{1,2}A_{1,2}\right)}\mathbf{y}_{E_2\left(A_{1,2}\right)} \end{bmatrix}$$

(equation 1.10).

However, although the vectors $\begin{bmatrix} \hat{\mathbf{u}}_{E_1\left(A^0_{1,2}\right)} \\ \hat{\mathbf{u}}_{E_1\left(A_2\right)} \end{bmatrix}$ and $\begin{bmatrix} \hat{\mathbf{u}}_{E_2\left(A^0_{1,2}\right)} \\ \hat{\mathbf{u}}_{E_2\left(A_1\right)} \end{bmatrix}$ remain unknown in practice, they could be predicted from the known vectors $\begin{bmatrix} \hat{\mathbf{u}}_{E_1\left(A_1\right)} \\ \hat{\mathbf{u}}_{E_1\left(A_{1,2}\right)} \end{bmatrix}$ and $\begin{bmatrix} \hat{\mathbf{u}}_{E_2\left(A_2\right)} \\ \hat{\mathbf{u}}_{E_2\left(A_{1,2}\right)} \end{bmatrix}$, e.g. through the selection index theory, respectively.

Therefore, the MME (1.1) can be written as

$$\left[\mathbf{G}_{E_0}^{-1} + \left(\mathbf{D}_{E_1}^{-1} - \mathbf{G}_{E_1}^{-1}\right) + \left(\mathbf{D}_{E_2}^{-1} - \mathbf{G}_{E_2}^{-1}\right)\right]\hat{\mathbf{u}}_J = \left[\mathbf{D}_{E_1}^{-1}\hat{\mathbf{u}}_{E_1} + \mathbf{D}_{E_2}^{-1}\hat{\mathbf{u}}_{E_2}\right] \text{ (equation 1.11).}$$

By replacing $\mathbf{G}^{*-1}$ in the equation 1.2 by the LHS of the equation 1.11 and $\mathbf{G}^{*-1}\boldsymbol{\mu}$ by the RHS of the equation 1.11, we obtain:

$$\begin{bmatrix} \mathbf{X'}_{E_0}\mathbf{R}_{E_0}^{-1}\mathbf{X}_{E_0} & \mathbf{X'}_{E_0}\mathbf{R}_{E_0}^{-1}\mathbf{Z}_{E_0} \\ \mathbf{Z'}_{E_0}\mathbf{R}_{E_0}^{-1}\mathbf{X}_{E_0} & \mathbf{Z'}_{E_0}\mathbf{R}_{E_0}^{-1}\mathbf{Z}_{E_0} + \mathbf{G}_{E_0}^{-1} + \left(\mathbf{D}_{E_1}^{-1} - \mathbf{G}_{E_1}^{-1}\right) + \left(\mathbf{D}_{E_2}^{-1} - \mathbf{G}_{E_2}^{-1}\right) \end{bmatrix} \begin{bmatrix} \hat{\boldsymbol{\beta}}_{E_0} \\ \hat{\mathbf{u}}_{E_0} \end{bmatrix} = \begin{bmatrix} \mathbf{X'}_{E_0}\mathbf{R}_{E_0}^{-1}\mathbf{y}_{E_0} \\ \mathbf{Z'}_{E_0}\mathbf{R}_{E_0}^{-1}\mathbf{y}_{E_0} + \mathbf{D}_{E_1}^{-1}\hat{\mathbf{u}}_{E_1} + \mathbf{D}_{E_2}^{-1}\hat{\mathbf{u}}_{E_2} \end{bmatrix}.$$

This development could be extended to more than two sources of external information.
