# Supplementary material for: Unified method to integrate and blend several, potentially related, sources of information for genetic evaluation
Source: Genet Sel Evol. 2014 Sep 30;46(1):59. doi: 10.1186/s12711-014-0059-3 (PMC4179859; doi:10.1186/s12711-014-0059-3)
Supplement: Additional file 2 — Double-counting between internal and external information. This file describes the development to avoid double-counting of contributions due to records between internal and external information. [file 12711_2014_59_MOESM2_ESM.pdf]

Additional file 2

Double counting between internal and external information

Assume $\hat{\mathbf{u}}_{\mathrm{E}_0}$ and $\mathbf{D}_{\mathrm{E}_0}^{-1} = \mathbf{G}_{\mathrm{E}_0}^{-1} + \mathbf{\Lambda}_{\mathrm{E}_0}$ (equation 2.1), the vector of known internal EBV and the inverse of the associated prediction error (co)variance matrix obtained from the genetic evaluation E_0 based on the source E_0 including only internal information where $\mathbf{G}_{\mathrm{E}_0}^{-1}$ is the inverse of the additive (co)variance matrix for all internal and external animals in the genetic evaluation E_0 and $\mathbf{\Lambda}_{\mathrm{E}_0}$ is a block diagonal variance matrix. The vector $\hat{\mathbf{u}}_{\mathrm{E}_1}$ and the matrix $\mathbf{D}_{\mathrm{E}_1}^{-1} = \mathbf{G}_{\mathrm{E}_1}^{-1} + \mathbf{\Lambda}_{\mathrm{E}_1}$ (equation 2.2) are the vector of known external EBV and the inverse of the associated prediction error (co)variance matrix obtained from a genetic evaluation based on the source E_1 including external and internal information where $\mathbf{G}_{\mathrm{E}_1}^{-1}$ is the inverse of the additive (co)variance matrix for all internal and external animals in the genetic evaluation E_1. The vector $\hat{\mathbf{u}}_{\mathrm{E}_2}$ and the matrix $\mathbf{D}_{\mathrm{E}_2}^{-1}$ are the vector of unknown external EBV and the inverse of the associated unknown prediction error (co)variance matrix obtained from a genetic evaluation E_2 based on the source E_2 including only external information. It is also assumed that double counting among animals due to relationships is taken into account.

Therefore, from $\mathbf{\Lambda}_{\mathrm{E}_0}$ and $\mathbf{\Lambda}_{\mathrm{E}_1}$, the diagonal matrix of RE expressing the amount of contributions only due to records, $\mathbf{RE}_{\mathrm{E}_0}$ and $\mathbf{RE}_{\mathrm{E}_1}$, can be estimated for the two sources of information E_0 and E_1, respectively. Because these RE are free of contributions due to relationships and due to correlated traits, the matrix of RE associated with the source of information E_2, $\mathbf{RE}_{\mathrm{E}_2}$, can be estimated as follows:

$$\mathbf{RE}_{\mathrm{E}_2} = \mathbf{RE}_{\mathrm{E}_1} - \mathbf{RE}_{\mathrm{E}_0} \qquad \text{(equation 2.3).}$$

It can be also written that $\mathbf{\Lambda}_{\mathrm{E}_2} = \mathbf{\Lambda}_{\mathrm{E}_1} - \mathbf{\Lambda}_{\mathrm{E}_0}$ (equation 2.4). The unknown $\mathbf{D}_{\mathrm{E}_2}^{-1}$ can be

approximated as $\mathbf{D}_{E_2}^{-1} = \mathbf{G}_{E_2}^{-1} + \mathbf{\Lambda}_{E_2}$ (equation 2.5) where $\mathbf{G}_{E_2}^{-1}$ is the inverse of an unknown additive (co)variance matrix for the external source E_2. From the equations 2.1, 2.2 and 2.5, the equation 2.4 is equivalent to the equation $\mathbf{D}_{E_2}^{-1} - \mathbf{G}_{E_2}^{-1} = (\mathbf{D}_{E_1}^{-1} - \mathbf{G}_{E_1}^{-1}) - (\mathbf{D}_{E_0}^{-1} - \mathbf{G}_{E_0}^{-1})$ (equation 2.5).

Following the equations (1) and assuming the lack of phenotypes in $\mathbf{y}_{\mathbf{E_0}}$, it can be written:

$$\left[\mathbf{G}_{E_1}^{-1} + \mathbf{D}_{E_0}^{-1} - \mathbf{G}_{E_0}^{-1} + \mathbf{D}_{E_2}^{-1} - \mathbf{G}_{E_2}^{-1}\right]\hat{\mathbf{u}}_{E_1} = \mathbf{D}_{E_0}^{-1}\hat{\mathbf{u}}_{E_0} + \mathbf{D}_{E_2}^{-1}\hat{\mathbf{u}}_{E_2} \quad (2.6).$$

Because of the equation 2.5, the equation 2.6 can be written as follows:

$$\mathbf{D}_{E_1}^{-1}\hat{\mathbf{u}}_{E_1} = \mathbf{D}_{E_0}^{-1}\hat{\mathbf{u}}_{E_0} + \mathbf{D}_{E_2}^{-1}\hat{\mathbf{u}}_{E_2}.$$

Thereby, $\hat{\mathbf{u}}_{E_2}$ can be estimated using $\mathbf{D}_{E_2}^{-1}\hat{\mathbf{u}}_{E_2} = \mathbf{D}_{E_1}^{-1}\hat{\mathbf{u}}_{E_1} - \mathbf{D}_{E_0}^{-1}\hat{\mathbf{u}}_{E_0}$ (equation 2.7).

Because the source E_2 is free of internal information E_0, it can be integrated into the internal evaluation through the system of equations (1) as follows:

$$\begin{bmatrix} \mathbf{X}'_{E_0}\mathbf{R}_{E_0}^{-1}\mathbf{X}_{E_0} & \mathbf{X}'_{E_0}\mathbf{R}_{E_0}^{-1}\mathbf{Z}_{E_0} \\ \mathbf{Z}'_{E_0}\mathbf{R}_{E_0}^{-1}\mathbf{X}_{E_0} & \mathbf{Z}'_{E_0}\mathbf{R}_{E_0}^{-1}\mathbf{Z}_{E_0} + \mathbf{G}_{E_0}^{-1} + \mathbf{D}_{E_2}^{-1} - \mathbf{G}_{E_2}^{-1} \end{bmatrix} \begin{bmatrix} \hat{\boldsymbol{\beta}}_{E_0} \\ \hat{\mathbf{u}}_{E_0} \end{bmatrix} = \begin{bmatrix} \mathbf{X}'_{E_0}\mathbf{R}_{E_0}^{-1}\mathbf{y}_{E_0} \\ \mathbf{Z}'_{E_0}\mathbf{R}_{E_0}^{-1}\mathbf{y}_{E_0} + \mathbf{D}_{E_2}^{-1}\hat{\mathbf{u}}_{E_2} \end{bmatrix} \quad \text{(equation 2.8).}$$

Due to the equations 2.5 and 2.7, $\mathbf{D}_{E_2}^{-1}$ and $\hat{\mathbf{u}}_{\mathbf{E_2}}$ must not be estimated explicitly and the system of equations 2.8 can be written as follows:

$$\begin{bmatrix} \mathbf{X}'_{E_0}\mathbf{R}_{E_0}^{-1}\mathbf{X}_{E_0} & \mathbf{X}'_{E_0}\mathbf{R}_{E_0}^{-1}\mathbf{Z}_{E_0} \\ \mathbf{Z}'_{E_0}\mathbf{R}_{E_0}^{-1}\mathbf{X}_{E_0} & \mathbf{Z}'_{E_0}\mathbf{R}_{E_0}^{-1}\mathbf{Z}_{E_0} + \mathbf{G}_{E_0}^{-1} + \left(\mathbf{D}_{E_1}^{-1} - \mathbf{G}_{E_1}^{-1}\right) - \left(\mathbf{D}_{E_0}^{-1} - \mathbf{G}_{E_0}^{-1}\right) \end{bmatrix} \begin{bmatrix} \hat{\boldsymbol{\beta}}_{E_0} \\ \hat{\mathbf{u}}_{E_0} \end{bmatrix}$$
$$= \begin{bmatrix} \mathbf{X}'_{E_0}\mathbf{R}_{E_0}^{-1}\mathbf{y}_{E_0} \\ \mathbf{Z}'_{E_0}\mathbf{R}_{E_0}^{-1}\mathbf{y}_{E_0} + \mathbf{D}_{E_1}^{-1}\hat{\mathbf{u}}_{E_1} - \mathbf{D}_{E_0}^{-1}\hat{\mathbf{u}}_{E_0} \end{bmatrix}$$

This development can be extended to integrate several sources of external information.
